# Phosphatase-Dead Myotubularin Ameliorates X-Linked Centronuclear Myopathy Phenotypes in Mice

**DOI:** 10.1371/journal.pgen.1002965

**Published:** 2012-10-11

**Authors:** Leonela Amoasii, Dimitri L. Bertazzi, Hélène Tronchère, Karim Hnia, Gaëtan Chicanne, Bruno Rinaldi, Belinda S. Cowling, Arnaud Ferry, Bruno Klaholz, Bernard Payrastre, Jocelyn Laporte, Sylvie Friant

**Affiliations:** 1 Department of Translational Medecine, Institut de Génétique et de Biologie Moléculaire et Cellulaire (IGBMC), INSERM U964, CNRS UMR7104, Université de Strasbourg, Collège de France, Illkirch, France; 2 Department of Molecular and Cellular Genetics, UMR7156, Université de Strasbourg and CNRS, Strasbourg, France; 3 INSERM, U1048 and Université Toulouse 3, I2MC, Toulouse, France; 4 UMRS974, Université Pierre et Marie Curie, Paris, France; 5 Department of Integrated Structural Biology, IGBMC, INSERM U964, CNRS UMR7104, Université de Strasbourg, Illkirch, France; 6 CHU de Toulouse, Laboratoire d'Hématologie, Toulouse, France; The Jackson Laboratory, United States of America

## Abstract

Myotubularin MTM1 is a phosphoinositide (PPIn) 3-phosphatase mutated in X-linked centronuclear myopathy (XLCNM; myotubular myopathy). We investigated the involvement of MTM1 enzymatic activity on XLCNM phenotypes. Exogenous expression of human MTM1 in yeast resulted in vacuolar enlargement, as a consequence of its phosphatase activity. Expression of mutants from patients with different clinical progression and determination of PtdIns3*P* and PtdIns5*P* cellular levels confirmed the link between vacuolar morphology and MTM1 phosphatase activity, and showed that some disease mutants retain phosphatase activity. Viral gene transfer of phosphatase-dead myotubularin mutants (MTM1^C375S^ and MTM1^S376N^) significantly improved most histological signs of XLCNM displayed by a *Mtm1*-null mouse, at similar levels as wild-type MTM1. Moreover, the MTM1^C375S^ mutant improved muscle performance and restored the localization of nuclei, triad alignment, and the desmin intermediate filament network, while it did not normalize PtdIns3*P* levels, supporting phosphatase-independent roles of MTM1 in maintaining normal muscle performance and organelle positioning in skeletal muscle. Among the different XLCNM signs investigated, we identified only triad shape and fiber size distribution as being partially dependent on MTM1 phosphatase activity. In conclusion, this work uncovers MTM1 roles in the structural organization of muscle fibers that are independent of its enzymatic activity. This underlines that removal of enzymes should be used with care to conclude on the physiological importance of their activity.

## Author Summary

X-linked centronuclear myopathy is a muscle disorder characterized by neonatal hypotonia and abnormal organelle positioning in skeletal muscle. This myopathy is due to different mutations in the MTM1 gene encoding the phosphoinositide phosphatase myotubularin. Disease-causing mutations are found all along the protein sequence and not only in the phosphatase catalytic domain. We investigated the link between myotubularin phosphatase activity and disease phenotypes. We used brewer yeast as a simple cellular model to analyze the *in vivo* phosphatase activity of different disease mutants. Our results show that mutations responsible for severe forms of myopathy are either active or inactive phosphatases. To further question this finding, we used the mice myotubularin knock-out model that reproduces faithfully the histopathological findings from human patients. Expression of phosphatase-dead mutants improved most phenotypes of knock-out mice comparable to wild-type myotubularin. This shows that the maintenance of normal skeletal muscles is largely independent from myotubularin phosphatase activity, while defects in the activity may participate in the onset of the disease. Moreover, it could have important implications in the design of therapeutic approaches aimed at manipulating the phosphoinositide levels in the different diseases linked to myotubularin homologues. Finally, these data call for cautiousness when manipulating such enzymes to conclude on the physiological relevance of their activity.

## Introduction

X-linked centronuclear myopathy (XLCNM, also called myotubular myopathy; OMIM 310400) is a recessive congenital muscle disorder affecting mainly males and due to mutations in the *MTM1* gene coding for the phosphoinositides (PPIn) phosphatase myotubularin [1]. The most severe form of XLCNM is characterised by hypotonia at birth, muscle atrophy, generalized muscle weakness and respiratory failure leading to high neonatal mortality [2]. Milder clinical phenotypes and progression were also reported and some are compatible with nearly normal lifespan [3]. Muscle biopsies from XLCNM patients show hypotrophic muscle fibers with an abnormal central positioning of nuclei. A mouse model lacking the MTM1 protein (*Mtm1* KO) has been characterized and reproduces the muscle mass decrease and most histopathological features of XLCNM, including muscle fibers hypotrophy and abnormal organelles positioning [4]. While the *MTM1* gene is ubiquitously expressed, skeletal muscle is the tissue mainly affected. To date almost 200 different disease-causing mutations have been identified in the *MTM1* gene [3], [5]–[7]. Most mutations cause severe forms of the myopathy characterized by a strong decrease in the protein level, at least in fibroblasts or lymphoblasts, whereas others cause milder forms of the disease [8], [9]. A very mild XLCNM phenotype was even described in a 67-year-old grandfather with a N180K missense mutation [3]. However, the genotype-phenotype correlation is not extensive and the importance of the PPIn phosphatase activity in the disease phenotype was not defined.

Myotubularin (MTM1) displays PPIn 3-phosphatase activity and converts phosphatidylinositol 3-phosphate (PtdIns3*P*) into PtdIns and phosphatidylinositol 3,5-bisphosphate (PtdIns(3,5)*P*_2_) into PtdIns5*P*. The PtdIns3*P* phosphatase activity of myotubularin was identified in a purified protein complex in brain and confirmed *in vitro* and *ex vivo* after the isolation of the cDNA [10], [11]. The catalytic site and mechanism of MTM1 resembles those of dual-specificity protein phosphatases. Indeed, mutation of the catalytic cysteine of MTM1 into serine (C375S, phosphatase-dead) totally abolished its enzymatic activity [12]–[14]. PtdIns3*P* produced by the PtdIns 3-kinase hVPS34/Vps34, is enriched at early and late endosomes and is essential for endosomal protein sorting and trafficking, autophagy and proper morphology of the endosomal compartment in human and yeast cells (for a review see [15]). PtdIns3*P* is also produced by class II PtdIns 3-kinases in multicellular eukaryotes, while these kinases are absent in yeasts [16], [17]. PtdIns(3,5)*P*_2_, the other substrate of MTM1, is generated from PtdIns3*P* by the 5-kinase PIKfyve/Fab1. The absence of PtdIns(3,5)*P*_2_ resulting from impairment of PIKfyve activity in mammalian cells, or from *FAB1* gene deletion in yeast *S. cerevisiae*, leads to a swollen or enlarged endosomal/lysosomal compartment associated with retrograde endosomal trafficking defects (for a review see [18]).

The study of MTM1 in human cells is hampered by the presence of highly conserved paralogues, termed MTMR for myotubularin related proteins. There are thirteen MTMR proteins (MTMR1 to MTMR13), seven of which are active phosphatases while the other six are dead phosphatases lacking key catalytic residues. *S. cerevisiae* contains only one member of the myotubularin family, Ymr1 (yeast myotubularin related-1) encoded by the *YJR110W* gene [19], [20]. Ymr1 displays PtdIns 3-phosphatase activity *in vitro* and *in vivo* and deletion of the *YMR1* gene leads only to minor phenotypes [12], [21].

The aim of this study is to understand the importance of the MTM1 PPIn phosphatase activity in the phenotype and severity of the disease. We first analyzed at the cellular level the functional impact of MTM1 mutations isolated from patients using yeast *S. cerevisiae ymr1Δ* deletion strains. Among the described mutations in *MTM1*, we have chosen to enzymatically characterize missense mutations affecting different MTM1 domains that lead to severe, mild or very mild XLCNM forms. We then analyzed the morphology of the vacuolar/lysosomal compartment, the subcellular distribution of these proteins and the intracellular levels of the different PPIn. The yeast results show that some disease mutants display phosphatase activity. In parallel, we performed rescue experiments for the XLCNM-like phenotypes displayed by the *Mtm1* KO mice using adeno-associated viral gene transfer of murine wild-type, phosphatase-inactive C375S or S376N constructs. Taken together, our data show that phosphatase-dead MTM1 mutants ameliorated most phenotypes of knock-out mice thus suggesting that myotubularin displays phosphatase-independent functions to maintain normal skeletal muscle.

## Results

### Expression of human Myotubularin MTM1 in yeast impairs vacuolar morphology

**Figure 1:**
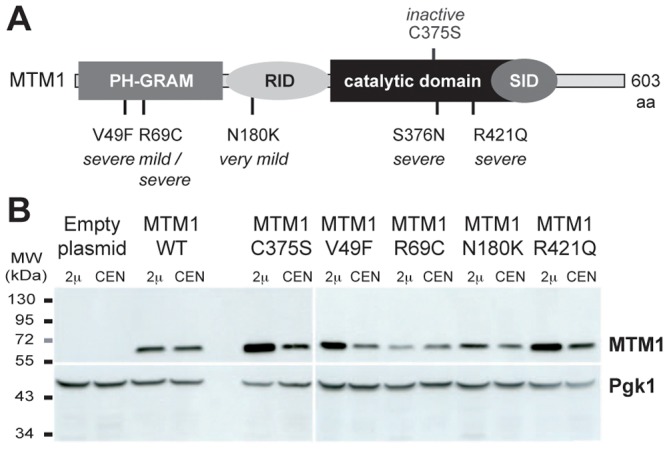
Human myotubularin expression in yeast *S. cerevisiae*. (A) Representation of the MTM1 protein with its domains, the position of the mutations analyzed and the severity of the resulting myopathy phenotype. MTM1 displays different domains, PH-GRAM (pleckstrin homology-glucosyltransferase, Rab-like GTPase activator and myotubularin), RID (Rac-induced recruitment domain), catalytic phosphatase domain and SID (SET-protein interaction domain). (B) Anti-MTM1 Western-blot on yeast protein extracts. The *MTM1* gene was placed under the control of the tetracycline-repressible *tetO* promoter in the low copy number centromeric plasmid (CEN, pVV204, 1 to 3 copies per cell). In the high copy number 2 µ plasmid (2 µ, pVV200, 20 to 50 copies per cell), the *MTM1* gene was under the control of the strong yeast *PGK1* promoter. These different plasmids were transformed into the *ymr1Δ* yeast mutant. Protein extracts of *ymr1Δ* cells transformed with pVV204 (CEN) or pVV200 (2 µ) empty plasmids (Control) or bearing the different MTM1 forms were analyzed by Western-blot. MTM1 production was detected with the mouse monoclonal 1G6 anti-MTM1 antibody. The different human MTM1 proteins were produced at the expected molecular weight (70 kDa) as compared to the *ymr1Δ* cells transformed with empty control plasmids that displayed no signal. Protein loading was evaluated by immunodetection of the yeast endogenous 3-phosphoglycerate kinase Pgk1 protein.

We analyzed in yeast *S. cerevisiae* the expression of four missense mutations in *MTM1*, two affecting the PPIn interaction PH-GRAM domain (MTM1^V49F^ and MTM1^R69C^), one the protein-protein interaction domain RID (MTM1^N180K^) and one the phosphatase catalytic domain (MTM1^R421Q^), leading respectively to severe, mild or severe (depending on the family), very mild, and severe XLCNM forms (Figure 1A) [3], [5], [6], [22]. We used as controls the wild-type MTM1 and the artificial phosphatase-dead MTM1^C375S^ mutant [12]. The different MTM1 constructs were expressed into the *ymr1Δ* yeast mutant from either low (CEN) or high (2 µ) copy number plasmids. Growth curves (optical density of liquid cultures as a function of time) as well as drop tests revealed that none of the plasmids induced a significant growth defect and that only cells expressing MTM1 or MTM1^R69C^ showed a slight growth delay (Figure S1). The Western blot analysis shows that the different human MTM1 proteins were produced at the expected molecular weight (70 kDa) and that protein levels in cells transformed with CEN plasmids were lower than in cells transformed with 2 µ plasmids (Figure 1B). Nonetheless, there were differences in the levels of the different MTM1 produced. Indeed, mutants having little or no effect on growth, like the phosphatase-dead MTM1^C375S^, were most abundant while mutants delaying growth, like MTM1^R69C^, were least abundant suggesting that yeast cells regulate the production of the exogenous human proteins.

**Figure 2:**
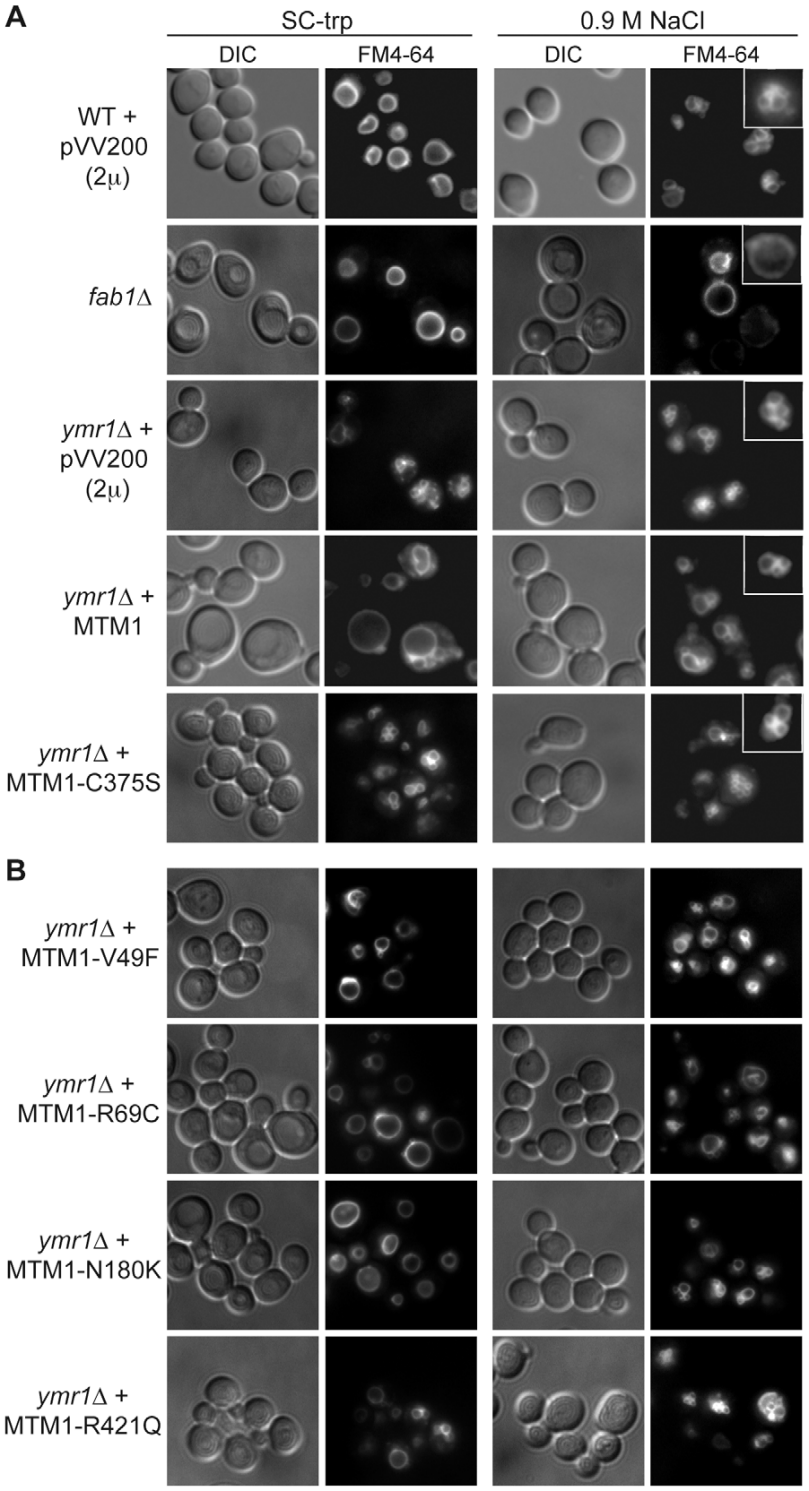
Yeast vacuolar phenotype analysis upon MTM1 expression. The wild-type and *fab1Δ* cells were used as controls. The different yeast cells transformed or not with pVV200 (2 µ, overexpression) plasmids bearing or not wild-type or mutants MTM1 were grown to exponential phase in selective SC-trp medium and the vacuoles were stained by FM4-64. Cell were labeled with FM4-64 for 15 min at 25°C in YPD and washed in once in SC-trp. Cells were then observed in selective medium by fluorescence microscopy with DIC (Nomarski) and TRITC (FM4-64) filters. Osmotic shock was induced by addition of NaCl (final concentration 0.9 M) to FM4-64 stained cultures, and cells were observed after 10 min incubation. (A) Wild-type, *fab1Δ* and *ymr1Δ* yeast cells transformed with pVV200 empty plasmid or bearing wild-type MTM1 or the phosphatase-dead MTM1^C375S^ mutant. Inset shows an increased magnification of a representative cell to illustrate the vacuolar phenotype upon osmotic shock. (B) *ymr1Δ* cells transformed with pVV200 plasmid bearing the different MTM1 mutants responsible for XLCNM myopathy.

**Figure 3:**
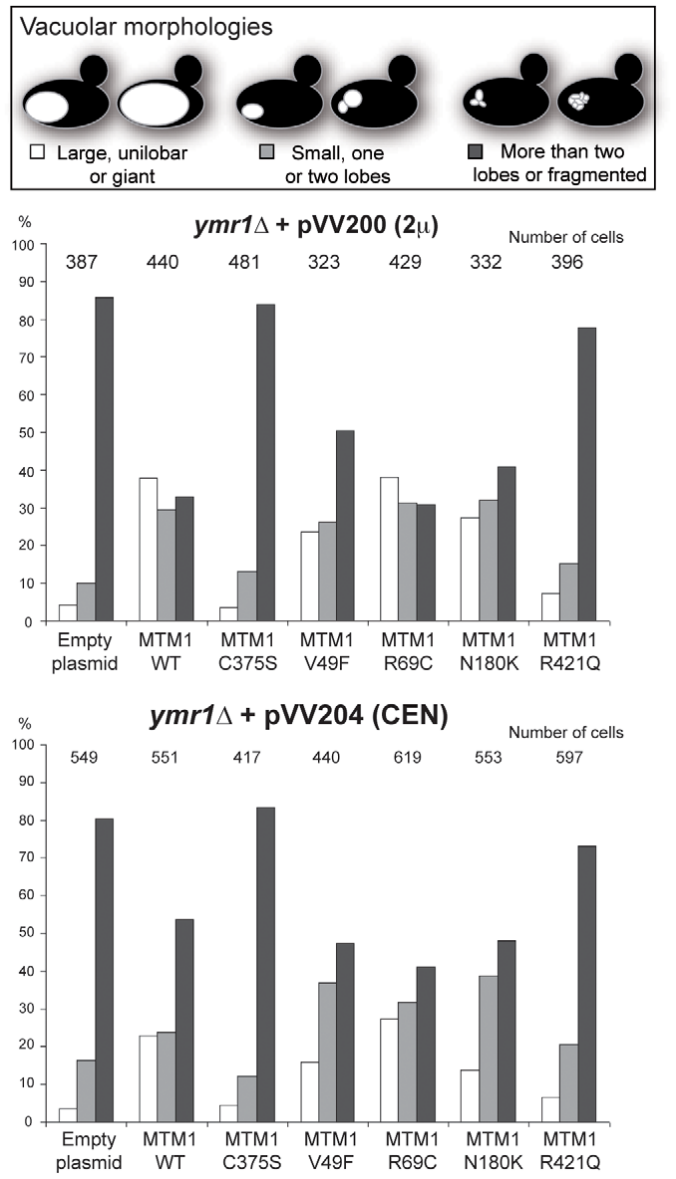
Vacuolar morphologies quantification in yeast cells producing MTM1. The *ymr1Δ* cells expressing either wild-type MTM1 or the different MTM1 mutants from either pVV200 (2 µ, overexpression) or pVV204 (CEN, expression) plasmid were analyzed. For each strain, 300 to 600 cells were observed by microscopy (DIC and FM4-64) and sorted into one of the three categories: unilobar large or giant (in white), small one or two lobes (in grey) and more than two lobes or fragmented (in black) vacuoles. The main vacuolar phenotype of the non-transformed *ymr1Δ* mutant cells is fragmented vacuoles with more than two lobes. Histograms are the mean of three independent experiments and show the proportion of each category in the different transformed yeast cells.

The expression of *MTM1* in fission yeast *Schizosaccharomyces pombe* induced an enlarged vacuolar phenotype [13], [23]. This was also observed upon expression of *MTMR3* in *S. cerevisiae* and the enlarged vacuolar phenotype was correlated with MTMR3 PPIn 3-phosphatase activity [24]. We stained the vacuolar membrane with the lipophilic fluorescent dye FM4-64 in *ymr1Δ* cells upon MTM1 or MTM1^C375S^ production (pVV204, CEN) or overproduction (pVV200, 2 µ). Wild-type yeast cells show unilobar vacuoles, whereas the *ymr1Δ* mutant displayed small multilobar vacuoles (Figure 2). In contrast, MTM1 overproduction resulted in larger cells with a small or large unilobar vacuole, whereas MTM1^C375S^ overproduction resulted in no obvious change in cell morphology (Figure 2A). The vacuolar morphologies were classified by microscopic observation into three categories: large unilobar or giant, small one or two lobes and more than two lobes or fragmented vacuoles (Figure 3). A significantly higher percentage of cells with abnormally large vacuoles was observed upon MTM1 production and this was increased with overproduction, whereas this percentage was low for MTM1^C375S^ and similar to the empty plasmid controls (Figure 3).

The enlarged vacuole phenotype could be due to the 3-phosphatase activity of MTM1 or to loss of Fab1 kinase functions upon MTM1 production. Indeed, the *fab1Δ* mutant also displays the enlarged vacuole phenotype (Figure 2A) [25]. Osmotic stress in wild-type yeast results in vacuolar fragmentation (Figure 2A), due to stimulation of Fab1 kinase activity [26], [27]. The *ymr1Δ* cells producing either MTM1 or phosphatase-dead MTM1^C375S^ displayed fragmented vacuoles upon osmotic shock, whereas in the *fab1Δ* mutant vacuoles remained enlarged (Figure 2A), indicating that overproduction of MTM1 did not block the Fab1 kinase activity. A similar result was previously obtained with *S. cerevisiae* cells expressing human *MTMR3* [24]. These results show that MTM1 phosphatase activity is directly responsible for the enlarged vacuole phenotype observed in yeast cells. Thus, analysis of vacuolar morphologies can be used to assess the MTM1 enzymatic activity of mutants found in XLCNM patients.

### Differential impact of XLCNM patient *MTM1* mutations on yeast vacuolar morphology

The vacuolar morphology of the *ymr1Δ* mutant expressing or overexpressing the four MTM1 constructs with XLCNM mutations was analyzed (Figure 1A). Many cells producing MTM1^V49F^, MTM1^R69C^ and MTM1^N180K^ displayed enlarged vacuoles (Figure 2B). Producing or overproducing the MTM1^R421Q^ in the *ymr1Δ* mutant did not result in major changes in vacuolar morphology, suggesting that this mutation strongly impairs the enzymatic activity *in vivo*. Upon production (CEN) and overproduction (2 µ) of MTM1^V49F^ or MTM1^N180K^, the percentage of cells with small and large unilobar vacuoles increased as observed for MTM1 (Figure 3). Thus MTM1^V49F^ and MTM1^N180K^ responsible for severe and very mild forms of XLCNM respectively display a phosphatase activity *in vivo*. Production (CEN and 2 µ) of MTM1^R69C^ resulted in similar or higher numbers of enlarged vacuoles as compared to MTM1 (Figure 3). As for MTM1, osmotic stress also induced fragmentation of the vacuole in cells expressing the different MTM1 with XLCNM mutations (Figure 2B) confirming that Fab1 kinase activity was not impaired. To determine whether similar enlarged vacuolar phenotypes were also observed in wild-type yeast cells (WT SEY6210) upon MTM1 expression, we analyzed the vacuolar morphology of WT cells transformed with pVV200 or pVV204 empty plasmids or coding for MTM1 or the different MTM1 mutants (Figure S2). The expression of the MTM1, MTM1^V49F^, MTM1^R69C^ and MTM1^N180K^ constructs induced an increase in the vacuolar size in both WT and *ymr1Δ* mutant cells, showing that this phenotype was not specific for the latter yeast mutant cells. Taken together, our data show that the vacuolar phenotypes induced by the different MTM1 mutants are not reflecting the severity of XLCNM phenotypes.

The enlarged vacuolar phenotypes observed upon production of various MTM1 proteins in yeast cells suggest that these human phosphatases have access to their membranous PPIn substrates. To determine their intracellular distribution, protein extracts from *ymr1Δ* cells producing the different MTM1 proteins were subjected to subcellular fractionation to separate the membrane fractions P13 and P100 from the cytosolic fraction S100 (Figure S3). MTM1 was found mainly in P13 and P100 fractions and a similar fractionation was observed for the different MTM1 mutants. This membrane association of MTM1, despite the absence of a transmembrane domain or a lipid anchor, suggests that MTM1 interacts with lipids or proteins independently of its phosphatase activity since MTM1^C375S^ was also membrane-associated. These results show that the different vacuolar phenotypes observed upon production of the different MTM1 proteins are not due to differences in their subcellular distribution.

### Some MTM1 disease mutants display phosphatase activity

**Figure 4:**
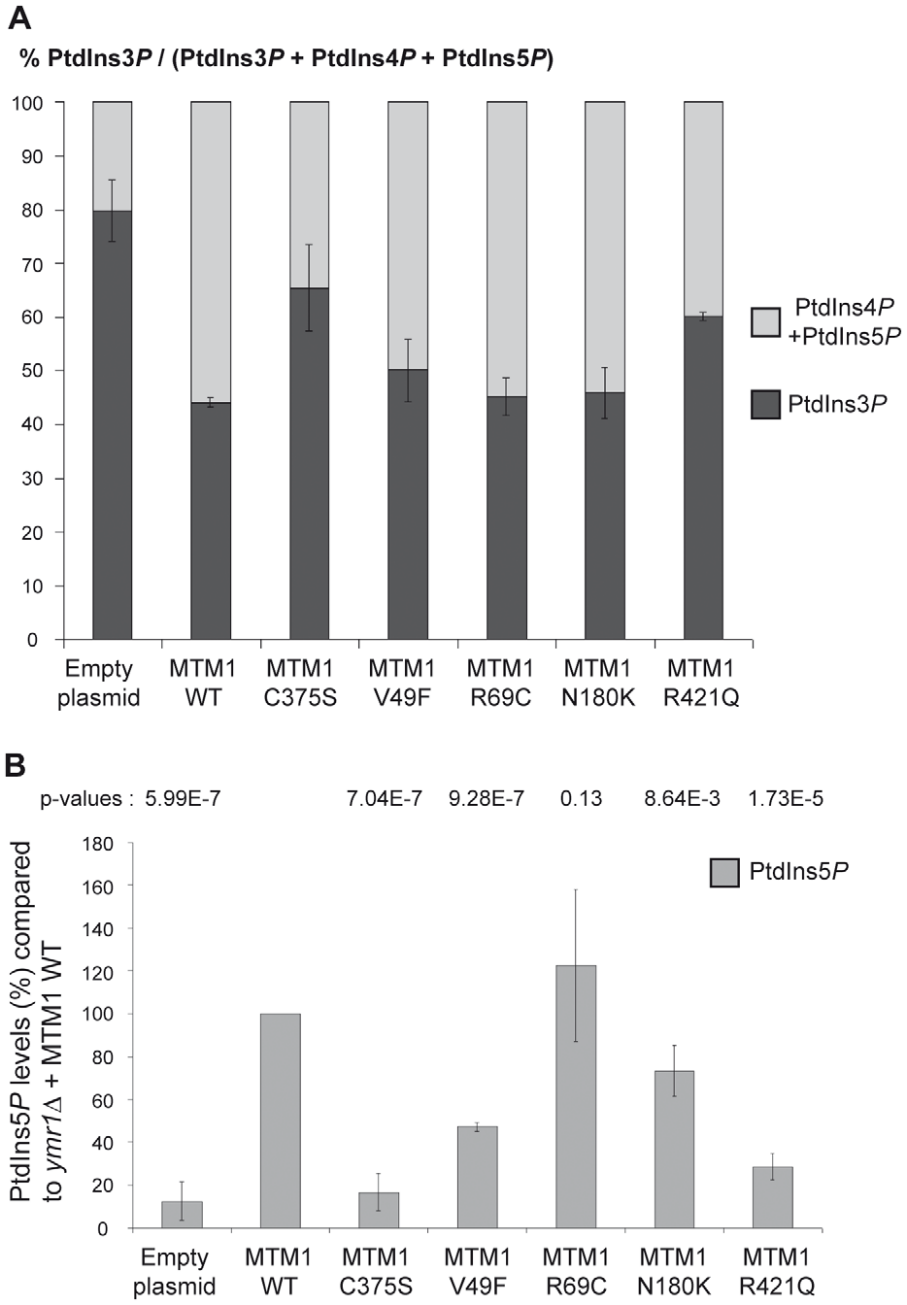
Determination of different phosphoinositides (PPIn) levels upon expression of MTM1 wild-type or mutant proteins. (A) Quantification of PtdIns3*P* cellular levels of *ymr1Δ* cells expressing wild-type MTM1 or the different mutants from pVV204 (CEN). Strains were grown to early log phase in selective medium, labeled with ^32^P and lipids were extracted and prepared for HPLC analysis. Based on HPLC chromatograms the peak area corresponding to each PPIn species was determined and results were expressed as the percentage of ^32^P-PtdIns3*P* compared to the total labeled ^32^P-PtdIns*P*. Results are represented as the mean of at least two independent experiments shown with standard deviations. (B) Quantitative analysis of PtdIns5*P* produced in *ymr1Δ* cells expressing wild-type or mutants MTM1. Strains were grown to log phase in selective medium, lipid were extracted, separated by TLC and spots corresponding to mono-phosphorylated PPIn were scrapped off and subjected to phosphorylation by PtdIns5*P* 4-kinase type IIα in presence of [γ-^32^P]-ATP. This kinase is specific for PtdIns5*P* and produces PtdIns(4,5)*P*_2_. The ^32^P-labeled PtdIns(4,5)*P*_2_ generated from this *in vitro* kinase reaction was further analyzed by TLC and radioactivity was quantified in a scintillation counter. The total amount of PtdIns5*P* (pmol) in each sample was determined by comparison with a calibration curve made from diC16-PtdIns5*P* and normalized to the number of yeast cells. Graph represents PtdIns5P as a percentage of production compared to the wild type myotubularin MTM1 (n = 3 to 4 experiments). The p-value for each construct was evaluated versus the wild type MTM1 and is indicated at the top of the graph. A p-value of less than 0.05 indicates that the difference in the two PtdIns5P percentages is statistically significant. The p-value was 0.0021 for MTM1^V49F^ versus MTM1^C375S^ and 0.0036 for MTM1^V49F^ versus MTM1^R421Q^.

The vacuolar phenotypes suggest that MTM1, MTM1^V49F^, MTM1^R69C^ or MTM1^N180K^ dephosphorylate PtdIns3*P* and PtdIns(3,5)*P*_2_ in yeast cells. To assess their phosphatase activity *in vivo* we determined the intracellular levels of PtdIns3*P* and PtdIns5*P* in *ymr1Δ* cells producing the different MTM1 constructs. Cells were labeled with ^32^P, lipids extracted and separated by thin-layer chromatography (TLC) and spots corresponding to phosphatidylinositol-monophosphates (PtdIns*P*) and phosphatidylinositol-bisphosphates (PtdIns*P*_2_) were isolated, deacylated and resolved by anion-exchange HPLC chromatography. Four different PPIn are identified in yeast *S. cerevisiae*: PtdIns3*P*, PtdIns4*P*, PtdIns(3,5)*P*_2_ and PtdIns(4,5)*P*_2_. The relative abundance of [PtdIns3*P*, PtdIns4*P*, PtdIns(3,5)*P*_2_, PtdIns(4,5)*P*_2_] is 40∶40∶7∶13 in wild-type SEY6210 strain whereas in *fab1Δ* mutant strain this ratio changes to 74∶21∶0∶5 [25]. It was previously shown that in *ymr1Δ* cells PtdIns3*P* levels are 2-fold higher than in wild-type cells and represent 82% of the total PtdIns*P* species [12]. To compare the phosphatase activity of the different MTM1 constructs, we calculated the percentage of PtdIns3*P* over total PtdIns*P* for the different strains (Figure 4A). This showed that MTM1^R421Q^ mutant affecting a residue in the catalytic pocket displayed a poor phosphatase activity, as PtdIns3*P* levels were comparable to those of the phosphatase-dead MTM1^C375S^ control. In contrast, the three other XLCNM patient mutants MTM1^V49F^, MTM1^R69C^ and MTM1^N180K^ showed PtdIns3*P* phosphatase activity comparable to the wild-type MTM1, as they displayed a strong decrease in the PtdIns3*P* levels which represented only 45–50% of total PtdIns*P* (Figure 4A). These results were confirmed by *in vitro* phosphatase assays [28] done on MTM1, MTM1^C375S^, MTM1^V49F^, MTM1^R69C^ and MTM1^N180K^ proteins immuno-isolated from yeast *ymr1Δ* cells (Figure S4).

Among the different PPIn detected in yeast cells, the PtdIns(3,5)*P*_2_ is the least abundant and represents about 0.1% of the total inositol phospholipids [18]. Indeed, HPLC chromatograms of PtdIns*P*_2_ showed that under our experimental conditions PtdIns(3,5)*P*_2_ was barely detectable in normal conditions for the different strains (not shown). PtdIns(3,5)*P*_2_ intracellular levels can be increased by osmotic shock [26]. To avoid any osmotic stress treatment of the cells and to detect PtdIns(3,5)*P*_2_ dephosphorylation by MTM1 under normal conditions, we quantified the resulting product PtdIns5*P* by a sensitive mass assay [28], [29]. Thus *ymr1Δ* yeast cells producing MTM1, MTM1^C375S^, MTM1^V49F^, MTM1^R69C^, MTM1^N180K^ and MTM1^R421Q^ were grown to exponential phase, lipids were extracted and separated by TLC, and spots corresponding to PtdIns*P* were extracted and submitted to an *in vitro* kinase assay to detect PtdIns5*P*. The *ymr1Δ* cells expressing MTM1^C375S^ or the empty plasmid showed a basal level of PtdIns5*P*, whereas in the presence of active MTM1 there was a strong increase in PtdIns5*P* (Figure 4B). Comparison of PtdIns5*P* levels showed that MTM1^R421Q^ can be considered as an inactive phosphatase, as it displayed similar levels to MTM1^C375S^ (Figure 4B). The three other XLCNM patient mutants MTM1^V49F^, MTM1^R69C^ and MTM1^N180K^ displayed a PtdIns(3,5)*P*_2_ phosphatase activity since significant quantities of PtdIns5*P* were detected (Figure 4B). Based on the p-values (Figure 4B), the PtdIns5*P* production by the MTM1^R69C^ mutant is not significantly different than the one detected for MTM1. These results were further confirmed by *in vitro* phosphatase assays showing proper dephosphorylation of PtdIns(3,5)*P*_2_ by MTM1, MTM1^V49F^, MTM1^R69C^ and MTM1^N180K^ produced in yeast *ymr1Δ* cells, whereas in the same conditions the MTM1^C375S^ and MTM1^R421Q^ were less active (Figure S4).

These results show that MTM1 mutants responsible for myopathy are either active or inactive phosphatases. Indeed, the MTM1^V49F^ mutant is associated to severe forms of the disease and displays phosphatase activity, even so its activity is reduced compared to the wild type phosphatase. The second mutant in the PH-GRAM domain of MTM1, MTM1^R69C^ shows similar phosphatase activity as the wild-type MTM1 and is associated to mild or severe phenotype. In conclusion, not all MTM1 mutants responsible for myopathy lack the phosphatase activity.

### Exogenous expression of myotubularin ameliorates the histological phenotype of *Mtm1* KO muscle independent of its enzymatic activity

**Figure 5:**
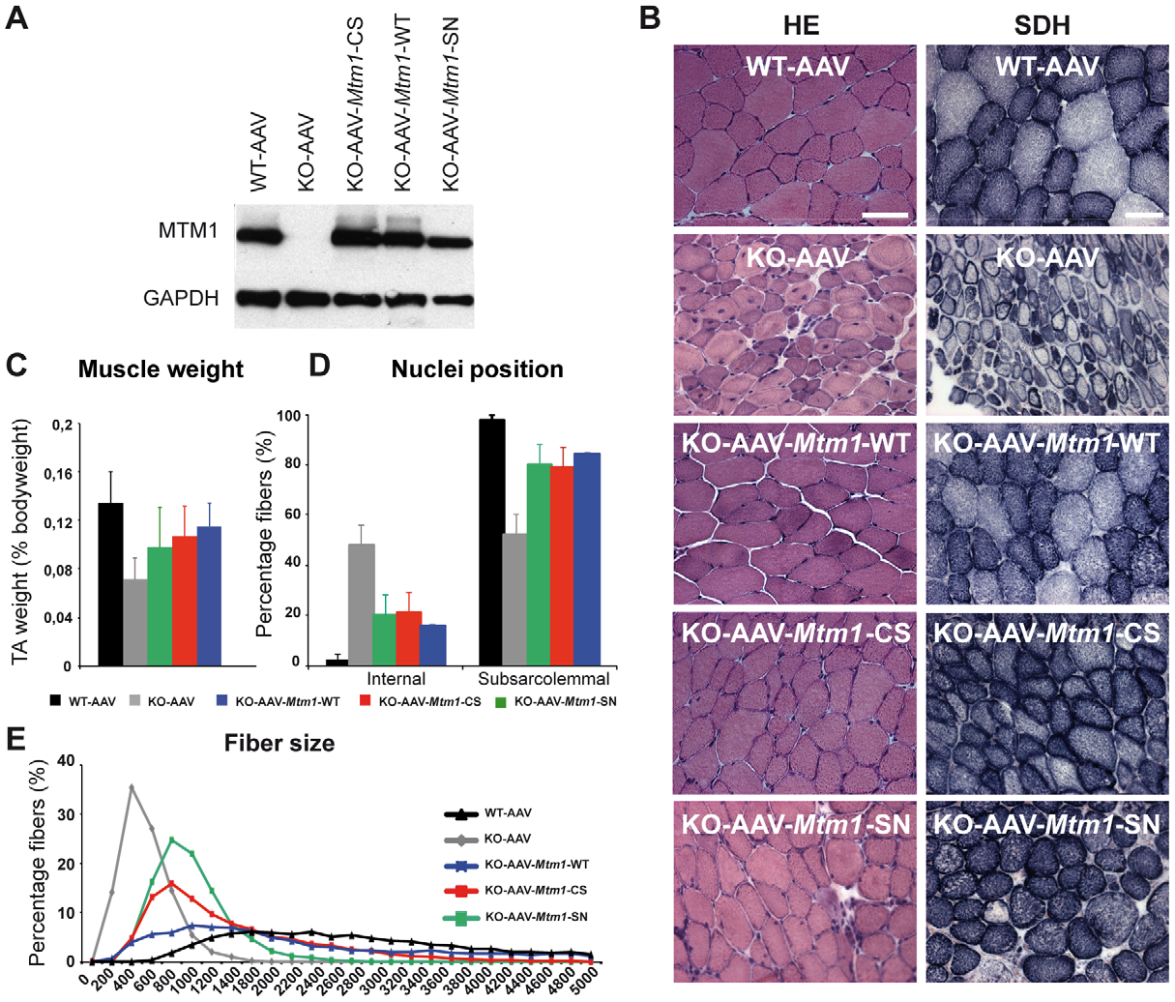
Amelioration of muscle atrophy and fiber hypotrophy in a murine model of centronuclear myopathy by AAV–mediated expression of two phosphatase-inactive myotubularin mutants MTM1^C375S^ and MTM1^S376N^. (A) Skeletal muscle protein lysates 4 weeks post-injection were immunoblotted for MTM1 and GAPDH levels in WT and *Mtm1* KO mice injected with empty AAV, *Mtm1* KO injected with AAV-*Mtm1*-WT, AAV-*Mtm1*-CS and AAV- *Mtm1*-SN. (B) Hematoxylin and eosin (HE, left panels, magnification ×400) and succinate dehydrogenase (SDH, right panels, ×400) staining of TA cross-sections from WT mice injected with empty AAV, *Mtm1* KO injected with empty AAV, AAV-*Mtm1*-WT, AAV-*Mtm1*-CS and AAV-*Mtm1*-SN mice 4 weeks PI. *Mtm1* KO muscle sections show the presence of very small myofibers and internalized nuclei. Mitochondrial oxidative staining is abnormally accumulated at the centre of fibers. Note the recovery of oxidative reactivity pattern in myotubularin-expressing *Mtm1* KO muscles. Scale bar: 50 µm. (C) Graph represents TA weight as a percentage of total body weight (n = 6 mice). P values<0.01 for WT injected with AAV versus KO injected with AAV, AAV-*Mtm1*-WT, AAV-*Mtm1*-CS and AAV-*Mtm1*-SN. P values<0.001 for KO injected with AAV versus AAV-*Mtm1*-WT, AAV-*Mtm1*-CS and AAV-*Mtm1*-SN. (D) Percentage of muscle fibers with internalized nuclei after AAV, AAV-*Mtm1*-WT, AAV-*Mtm1*-CS and AAV-*Mtm1*-SN injection of *Mtm1* KO mice. Nuclei were considered as internalized if not in contact with the sarcolemma. The number of fibers with internal nuclei is increased in *Mtm1* KO tibialis anterior (TA) muscle and significantly and equally reduced after injection with AAV-*Mtm1*-WT or AAV-*Mtm1*-CS or AAV-*Mtm1*-SN (n = 550). P values<0.009 for WT injected with AAV versus KO injected with AAV, AAV-*Mtm1*-WT, AAV-*Mtm1*-CS and AAV-*Mtm1*-SN. P values p<0.0009 for KO injected with AAV versus AAV-*Mtm1*-WT, AAV-*Mtm1*-CS and AAV-*Mtm1*-SN. (E) Transverse muscle sections were analyzed for fiber area. Fiber size is grouped into 200 µm^2^ intervals, and represented as the percentage of total fibers in each group (n = 1,000 for 15 mice for AAV-*Mtm1*-WT, AAV-*Mtm1*-CS and 6 mice for AAV-*Mtm1*-SN group).

As results in yeast suggested that some XLCNM patient mutants retain the phosphatase activity, we aimed to investigate the role of the phosphatase activity on the development of the XLCNM phenotype *in vivo*. We tested the ability of the MTM1^C375S^ phosphatase-dead mutant to correct the XLCNM-like muscle phenotype of *Mtm1* knockout (KO) mice compared to wild-type MTM1 using Adeno-associated virus (AAV) gene transfer. We used the constitutive *Mtm1* KO mouse that develops a homogeneous XLCNM in the 129PAS background [30]. These mutant animals show a progressive muscle weakness starting clinically at 3 weeks of age and leading to death by 7 to 9 weeks, probably from respiratory failure. They display most phenotypes observed in patients as a decrease in muscle mass, muscle fiber hypotrophy, nuclei and mitochondria positioning defects, desmin aggregation, and alteration in T-tubule structure. At 6 weeks old, *Mtm1* KO mice injected with empty AAV vector show a 38% decrease in the Tibialis anterior (TA) muscle weight compared to wild-type mice injected with empty AAV (Figure 5C). *Mtm1* KO TA muscles displayed smaller and rounder myofibers with increased proportion of internal nuclei compared to wild-type mice (Figure 5B and 5D). In addition, *Mtm1* KO muscles had an abnormal oxidative staining with higher intensities in the subsarcolemmal region and in the center of fibers, reminiscent of an accumulation of mitochondria at these regions (Figure 5B).

TA muscles of 2–3 weeks old *Mtm1* KO mice were injected with either AAV2/1-*Mtm1*-WT (AAV-*Mtm1*-WT) or AAV2/1-*Mtm1*-C375S (AAV-*Mtm1*-CS), where the C375S mutation abolishes the phosphatase enzymatic activity towards PPIn substrates (Figure 4). The contralateral muscle was injected with AAV2/1-Empty (AAV) as an internal control. The effect of AAV-mediated *Mtm1*-WT or *Mtm1*-CS expression was analyzed 4 weeks after injection. The level of ectopic *Mtm1*-WT and *Mtm1*-CS expression was analyzed by western blot in injected muscles. In both injected muscles the level of the protein was reestablished and similar to the levels of the endogenous protein in WT muscle (Figure 5A).

We confirmed that exogenous expression of *Mtm1*-WT in TA muscle corrects the XLCNM-like phenotype in the constitutive 129PAS *Mtm1* KO mice, as previously shown in muscle-specific KO mice on a B6 background [31], showing that the MTM1 protein is acting primarily in skeletal muscle and not in other tissues as only muscle was injected. The *Mtm1* KO model displays a severe muscle atrophy (Figure 5 and [4], [30]). Muscles injected with AAV-*Mtm1*-CS showed a significant increase of the weight compared to *Mtm1* KO muscle injected with AAV (0,13%±0,03 for AAV-*Mtm1*-CS compared to 0,08%±0,02 for AAV alone), reaching similar levels to *Mtm1* KO injected with *Mtm1*-WT (Figure 5C). Next we investigated if the increase in weight correlates with an improvement at the histological level. Hematoxylin and eosin (HE) staining revealed similar improvement of the histological aspects of *Mtm1* KO muscles injected with either AAV-*Mtm1*-WT or AAV-*Mtm1*-CS (Figure 5B). Quantitative analysis of the distribution of myofiber areas showed a clear increase in fiber size for both AAV-*Mtm1*-WT and AAV-*Mtm1*-CS treated muscles compared to *Mtm1* KO muscles (Figure 5E). None of the constructs restored fiber area and the muscle weight to the level of wild-type mice 4 weeks after infection and it was not possible to test if longer infection would increase the correction of these features under our experimental conditions as mice were dying around the age of analysis. Fiber area distribution is different comparing AAV-*Mtm1*-WT and AAV-*Mtm1*-CS; AAV-*Mtm1*-CS leads to a higher increase in the number of fibers with an area in the range of 200 to 3200 µm^2^, compared to fibers with an area superior to 3200 µm^2^ with AAV-*Mtm1*-WT. Our data show that both AAV-*Mtm1*-WT and AAV-*Mtm1*-CS partially but significantly improved muscle atrophy and fiber hypotrophy of the *Mtm1* KO mice.

Furthermore *Mtm1* KO muscle fibers are also characterized by a progressive disorganization in the distribution of mitochondria. Thus, we evaluated the localization of these organelles by succinate dehydrogenase staining (SDH) that labels the oxidative activity. The SDH staining of muscle sections (Figure 5B) revealed that the abnormal central concentration of oxidative activity was improved with both AAV-*Mtm1*-WT and AAV-*Mtm1*-CS. In addition, abnormal internalization of nuclei represents another hallmark of the XLCNM pathology. We thus counted the number of fibers with internal nuclei (not in contact with the sarcolemma) in wild-type and *Mtm1* KO muscles injected with AAV versus *Mtm1* KO muscles treated with AAV-*Mtm1*-WT or AAV-*Mtm1*-CS (Figure 5D). We observed a strong and similar reduction of the percentage of internal nuclei in AAV-*Mtm1*-WT and AAV-*Mtm1*-CS treated muscles compared to *Mtm1* KO muscles (21,15%±8,23 for AAV-*Mtm1*-CS; 15,68%±0,62 for AAV-*Mtm1*-WT compared to 46,67%±8,18 for *Mtm1* KO and 2,28%±2,11 for wild-type mice).

### The phosphatase-dead myotubularin improves muscle strength

**Figure 6:**
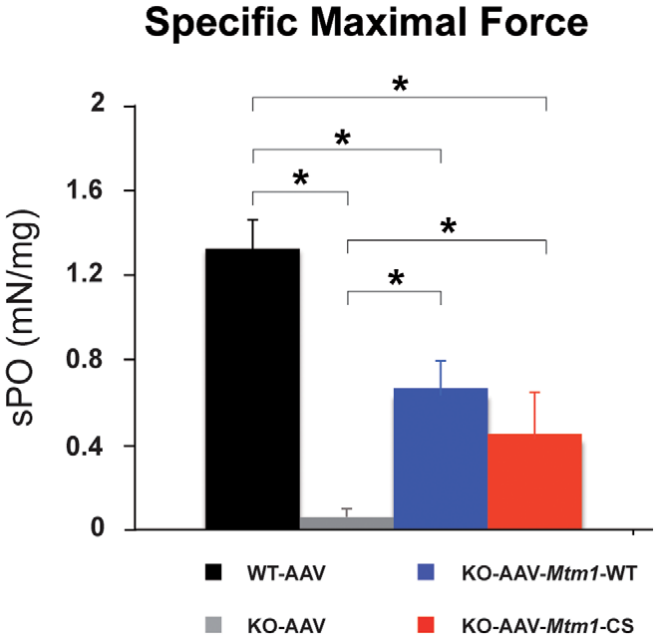
The phosphatase-dead C375S myotubularin mutant improves muscle force in *Mtm1* KO mice. The specific maximal force (sP0) of WT muscle injected with AAV and *Mtm1* KO TA muscle injected with AAV, AAV-*Mtm1*-WT and AAV-*Mtm1*-CS. The sP0 represents the absolute maximal force related to muscle weight (n = 6 mice, *P<0.001).

To determine whether the significant improvement of the histological features was associated to improved muscle performance, we measured the *in situ* force of the muscle. The isolated muscle was stimulated by the sciatic nerve, and the maximal force produced was recorded and normalized to muscle weight (Figure 6). The specific maximal force of untreated TA muscles of 6 week-old *Mtm1* KO mice was lower by 81% compared to wild-type muscle. The muscles transduced with AAV-*Mtm1*-CS and AAV-*Mtm1*-WT exhibit an increase of the specific maximal force compared to *Mtm1* KO (0,44 mN/mg±0,23 for AAV-*Mtm1*-CS; 0,66 mN/mg±0,17 for AAV-*Mtm1*-WT compared to 0,06 mN/mg±0,04 for *Mtm1* KO and 1,31 mN/mg±0,21 for wild-type mice) (Figure 6). Altogether, our results show that the MTM1^C375S^ phosphatase-dead mutant improves most XLCNM-like histological and the specific muscle force of the *Mtm1* KO model at a level comparable to that of the wild-type MTM1 protein.

### MTM1^C375S^ phosphatase-dead mutant restores the abnormal desmin organization in *Mtm1*-deficient muscle

**Figure 7:**
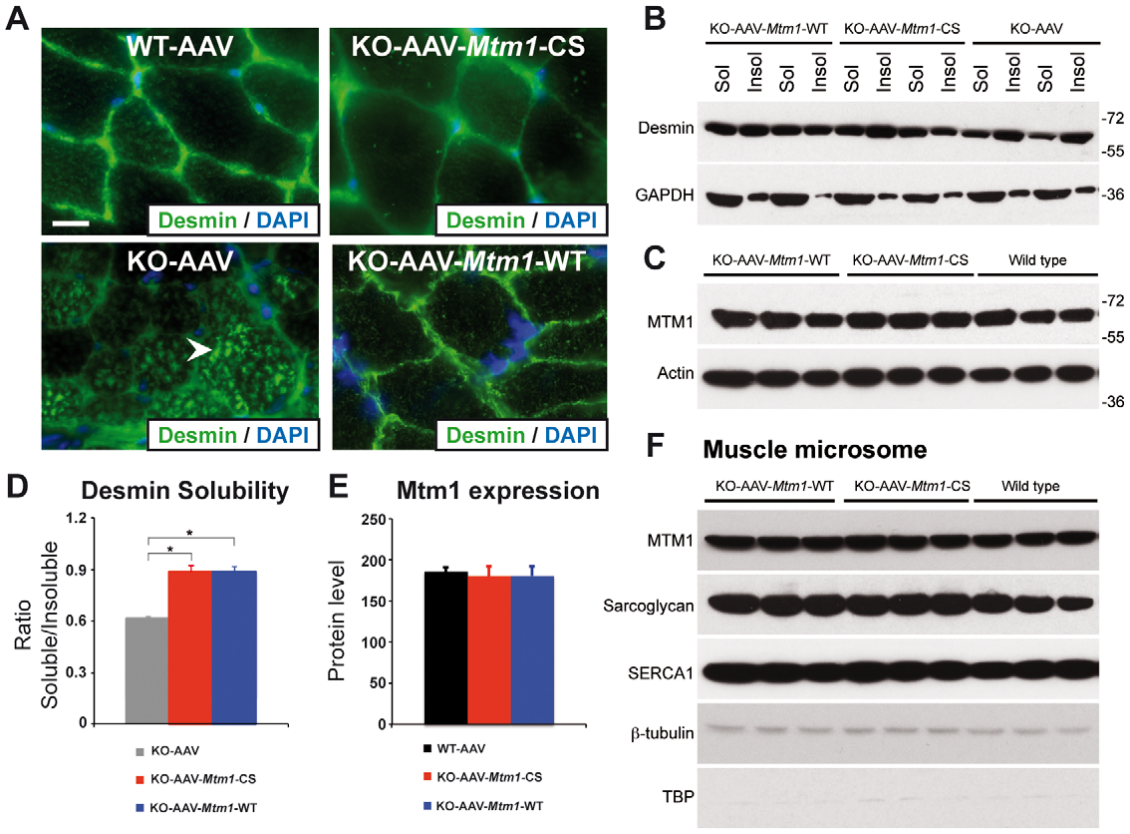
The phosphatase-dead C375S myotubularin mutant injection in *Mtm1*-KO muscle restores normal desmin expression and localization. (A) Ectopic expression of MTM1 transgene in *Mtm1*-KO muscle restored normal desmin localization in muscle. Arrowheads indicate aggregates of desmin in *Mtm1*-KO muscle injected with AAV. Scale bars: 10 µm. (B) The phosphatase-dead C375S myotubularin mutant expression in *Mtm1*-KO muscle restored normal desmin expression level in soluble and insoluble fraction. (C) Skeletal muscle protein lysates 4 weeks post-injection were immunoblotted for MTM1. (D) Quantification of relative desmin expression level in soluble compared to insoluble fraction. Data correlated from 2 independent experiments (n = 3 mice per group). *P≤0.05. (E) Quantification of relative expression of MTM1 in WT and *Mtm1* KO mice injected with empty AAV, *Mtm1* KO injected with AAV-*Mtm1*-WT and AAV-*Mtm1*-CS. (F) Microsome fractions from *Mtm1* KO muscles injected with AAV-*Mtm1-WT* and AAV-*Mtm1*-CS and from wild-type muscles were prepared and immunoblotted for MTM1 to compare localization of MTM1-WT and MTM1-C375S in the membrane fraction of the muscle. Microsome fractions were immunoblotted with antibodies detecting membrane proteins (SERCA1 and Sarcoglycan), cytoplasmic protein (β-tubulin) and nuclear protein (TATA-box binding protein (TBP)).

To further decipher the molecular basis for these phosphatase-independent improvements we analyzed the localization of desmin, a muscular MTM1 protein interactor. Indeed, it was recently shown that MTM1 binds specifically desmin and regulates the filament assembly and organization [32]. Desmin is the major component of intermediate filaments (IFs) cytoskeleton of muscle, which plays a central role in the integration of structure and function of striated muscle by linking the contractile apparatus to the sarcolemmal cytoskeleton as well as to several cytoplamic organelles and the nucleus. Desmin is found mainly in the Z-disk in a normal skeletal muscle. In the muscle biopsies from XLCNM patients, desmin localization is altered. The *Mtm1* KO mice muscles present an accumulation of aggregates that disrupt the continuity and organization of the desmin network (Figure 7A). We could previously show that ectopic expression of MTM1-WT in the *Mtm1* KO muscle restores the normal organization of the desmin network [32]. We examined the muscle injected with the MTM1^C375S^ phosphatase-dead mutant. The muscle transduced with AAV-*Mtm1*-CS exhibited a clear improvement of the desmin localization compared to the desmin aggregates observed in the *Mtm1* KO muscle injected with the empty virus (Figure 7A). Furthermore, the mislocalization of desmin in *Mtm1* KO corresponds also to a shift in desmin equilibrium from the soluble to the insoluble fraction, indicating a defect in the desmin assembly process (Figure 7B). We observed a significant and similar increase in the desmin solubility in AAV-*Mtm1*-WT and AAV-*Mtm1*-CS treated muscles compared to *Mtm1* KO muscles (Figure 7B). To confirm that AAV-*Mtm1*-WT and AAV-*Mtm1*-CS displayed similar efficiency regarding the correction of desmin solubility we analyzed the level of MTM1 expression in the same samples (Figure 7C). The MTM1 expression was similar in both type of muscles suggesting that phosphatase-dead mutant MTM1^C375S^ improves the desmin organization as efficiently as MTM1-WT. Moreover, we analyzed the distribution of MTM1-WT and MTM1-C375S in the membrane fraction of skeletal muscle using a microsomal preparation from *Mtm1* KO muscles injected with AAV-*Mtm1*-WT and AAV-*Mtm1*-CS and from wild-type muscle. Microsomal fractions were analyzed using protein markers for the membrane fraction (α-sarcoglycan and SERCA1), for the cytoplasmic (β-tubulin) and for the nuclear fractions (the TATA-box binding protein (TBP)). The MTM1-WT and MTM1-C375S proteins ectopically expressed in the *Mtm1*-KO muscle were similarly distributed in the microsomal fractions (Figure 7F). These results suggest that MTM1-WT and MTM1-C375S localize similarly in the membrane fraction in skeletal muscle.

Altogether, our results show that the phosphatase activity of MTM1 is not required for normal desmin localization in muscle fibers, and suggest that maintenance of the desmin network is a phosphatase-independent function of MTM1 that is important in XLCNM.

### Improvement of the ultrastructural organization of triads in *Mtm1* KO muscles

**Figure 8:**
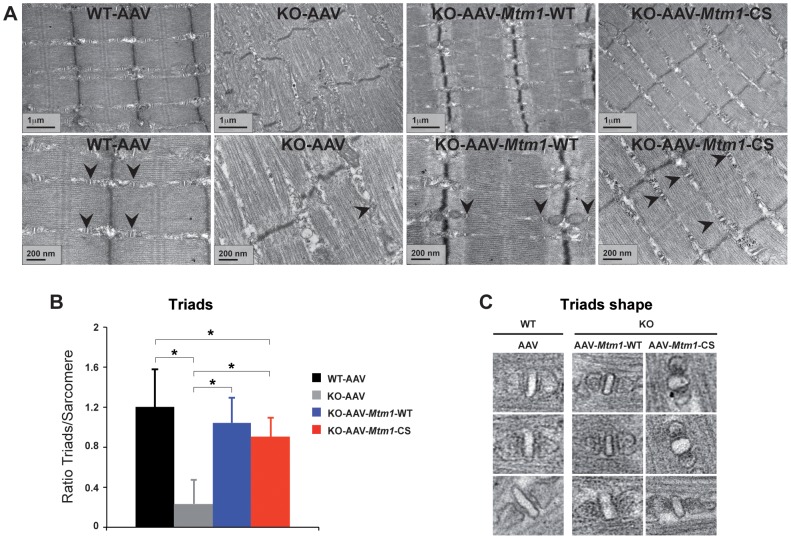
Improvement of triad abnormalities present in *Mtm1*-deficient muscles with AAV-*Mtm1*-WT and AAV-*Mtm1*-CS. (A) Sarcomere and triad (arrowheads) organization in wild-type muscle, *Mtm1* KO muscle, *Mtm1* KO muscles injected with AAV-*Mtm1*-WT and AAV-*Mtm1*-CS at 2 different magnifications. Muscles from *Mtm1* KO demonstrate a severe disorganization of the muscle fiber with lack of recognizable triads within the sarcomere structure. (B) Quantification of the presence of triads in the muscle fibers. The graph represents the ratio between the number of triads observed in each longitudinal section divided by the total number of sarcomeres present in the section. (C) Triads shape in wild-type muscle, and *Mtm1* KO muscles injected with AAV-*Mtm1*-WT and AAV-*Mtm1*-CS.

To determine whether the substantial amelioration of the histological features was correlated to the improvement of the structural organization of the triads, we analyzed the muscles by electron microscopy. Indeed, previous studies have shown that the muscles lacking myotubularin as well as XLCNM muscle biopsies present abnormal organization of triads [30], [33], [34]. The electron micrographs obtained from the wild-type, *Mtm1* KO and *Mtm1* KO injected with AAV-*Mtm1*-WT and AAV-*Mtm1*-CS muscles were analyzed. The wild-type muscle showed proper organization of the fibers and sarcomere arrangement, and the typical triad structure. In contrast, the micrographs from *Mtm1* KO muscle exhibited sarcomere disorganization and a decrease in the number of well-positioned triads (Figure 8A and 8B). Interestingly, the *Mtm1*-KO muscle injected with AAV-*Mtm1*-WT showed a clear improvement in the general organization of the sarcomere and the presence of the well–formed triads (Figure 8A–8C). An improvement was also observed in the *Mtm1* KO muscle injected with the AAV-*Mtm1*-CS. The ratio of triads per sarcomere in *Mtm1* KO muscles of 6 weeks-old mice was decreased by 83% compared to wild-type muscle. Muscles transduced with AAV-*Mtm1*-WT and AAV-*Mtm1*-CS presented a significant increase in this ratio (0,9±0,18 for AAV-*Mtm1*-CS; 1,04±0,24 for AAV-*Mtm1*-WT compared to 0,23±0,21 for *Mtm1* KO and 1,2±0,3 for wild-type mice) (Figure 8B). In addition, the shape of the triad was analyzed in the wild-type and in the *Mtm1*-KO muscle transduced with AAV-*Mtm1*-WT and AAV-*Mtm1*-CS. The *Mtm1* KO was not considered for this analysis, as recognizable triads were nearly absent in this muscle. The muscle transduced with the phosphatase-dead mutant exhibited recognizable triads with a more dilated shape than the muscle transduced with the AAV-*Mtm1*-WT and wild-type muscle (Figure 8C).

Altogether, our results show that the MTM1^C375S^ phosphatase-dead mutant improves the general organization of the muscle fibers and restores the presence and number of the triads in the *Mtm1* KO model at a level comparable to the wild-type MTM1 protein. However the muscle transduced with AAV-*Mtm1*-CS exhibited only a partial correction of the shape of the triads compared to the AAV-*Mtm1*-WT, suggesting a potential role of the phosphatase activity in the shape of the triad.

### The phenotypic improvement does not correlate with normalization of PtdIns3*P* levels

**Figure 9:**
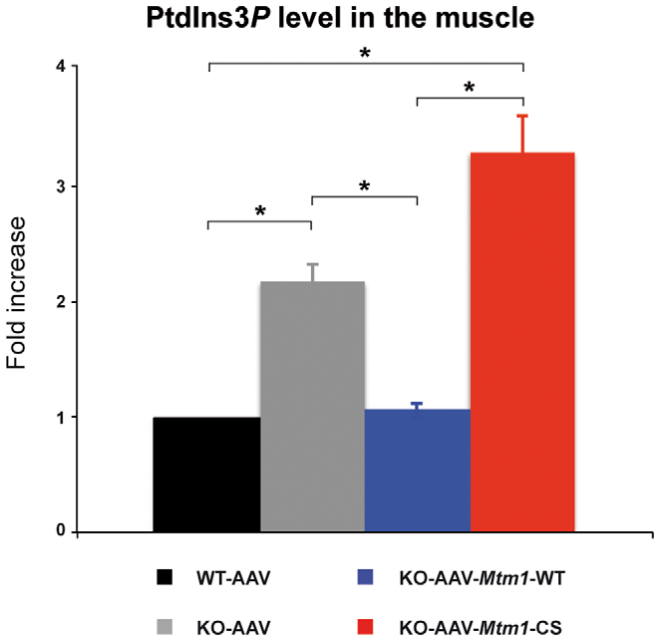
MTM1-WT but not MTM1-C375S normalizes PtdIns3*P* levels in the injected muscles. Lipids were extracted from wild-type, *Mtm1* KO, and *Mtm1*-KO injected with AAV-*Mtm1*-WT and AAV-*Mtm1*-CS tibialis anterior muscles and PtdIns3*P* levels were quantified. The results were presented as the levels of PtdIns3*P* to the total phospholipids. The graphs represent the mean of two independent experiments shown with the standard deviation. *p<0.05.

To determine whether the MTM1 phosphatase activity contributed to the improvement of the XLCNM phenotypes, we measured the level of PtdIns3*P* in the different muscles. For these measurements, we extracted total lipids from the tibialis anterior of wild-type, *Mtm1*-KO and *Mtm1*-KO muscles injected with AAV-*Mtm1*-WT or AAV-*Mtm1*-CS. We used a novel sensitive mass assay for measuring PtdIns3*P* from total muscle lipid extracts without metabolic labeling [35]. The level of PtdIns3*P* in the sample was quantified and normalized to total phospholipids, the resulting pmol of PtdIns3*P*/µmol of phospholipids data were expressed as fold increase compared to the wild-type muscle transduced with AAV (Figure 9). The lipid extracts from *Mtm1* KO mice exhibited a higher level of PtdIns3*P* compared to wild-type (2.19 fold increase for *Mtm1* KO muscle). These data support the conclusion that PtdIns3*P* is a physiological substrate of MTM1 in mammalian muscle and that the disease is paralleled by an alteration of PPIn metabolism in the *Mtm1* KO model. The lipid extracts from the muscles transduced with AAV-*Mtm1*-WT showed a normalization of the PtdIns3*P* to levels similar as the wild-type muscle. In contrast, muscles transduced with AAV-*Mtm1*-CS exhibited, as the *Mtm1* KO muscles, higher levels of Ptdns3*P* compared to the wild-type muscle (3.3 fold increase for AAV-*Mtm1*-CS) (Figure 9). The difference in PtdIns3*P* levels between the *Mtm1* KO injected with empty AAV and AAV-*Mtm1*-CS is not statistically significant since the p-value is 0.06. However, there is a tendency towards increased PtdIns3*P* levels with AAV-*Mtm1*-CS that could be caused by a substrate-trapping property of this mutant resulting in the protection of PtdIns3*P* from consumption by other enzymes. Thus, the *Mtm1*-C375S mutant is catalytically inactive *in vivo* and might be a substrate-trapping mutant. The results show that the correction of the phenotypes with the MTM1^C375S^ phosphatase-dead was not correlated to normalization of the PtdIns3*P* levels in muscle.

### The rescuing potential of the phosphatase-dead mutant is very likely not due to substrate-trapping properties

The analysis of the PtdIns3*P* level in mice muscles did not exclude that the MTM1^C375S^ mutant might be a substrate-trapping mutant. Thus, this mutant could promote the correction of the *Mtm1* KO mice phenotypes through a dominant-negative effect by blocking the access of effectors to PtdIns3*P* and/or PtdIns(3,5)*P*_2_. To address this issue, we used the MTM1^S376N^ (*Mtm1*-SN) mutant associated to severe XLCNM [36]. The S376N mutation located in the catalytic site abrogates the *in vitro* phosphatase activity [12] and this inactive mutant was predicted to disrupt the substrates binding based on the myotubularin MTMR2 crystal structure [37]. Indeed, the replacement of this serine 376 with a bulkier aminoacid removes the hydrogen bond formed with the oxygen of the D1 phosphate of the lipid substrate and is also predicted to produce an allosteric clash with both the D1 phosphate of the substrate and with two aminoacids of the catalytic pocket (Asp280 and Asp288, Figure S5). To determine its *in vivo* activity, we produced this mutant in yeast *ymr1Δ* cells, analyzed the vacuolar size and quantified the resulting PtdIns5*P* product (Figure S5). The results show that the MTM1^S376N^ protein is produced in yeast cells, and that this MTM1^S376N^ mutant is catalytically inactive as judged from the vacuolar phenotypes and the lack of dephosphorylation of PtdIns(3,5)*P*_2_ in PtdIns5*P* (Figure S5). Next, we analyzed the major XLCNM-like phenotypes in the *Mtm1* KO muscle injected with AAV-*Mtm1*-SN mutant (Figure 5). The *Mtm1* KO muscles transduced with AAV-*Mtm1*-CS showed a clear improvement of the muscle concerning the muscle weight, fiber size, organelle and nuclei positioning and a similar improvement was observed for the injection of AAV-*Mtm1*-SN mutant (Figure 5). While not excluding that the C375SS mutant could have some substrate-trapping properties, these results strongly suggest that the amelioration of the XLCNM phenotypes described for AAV-*Mtm1*-CS are not due to a substrate-trap effect of the C375S mutation. As the S376N mutant is also phosphatase-dead, this supports the conclusion that the MTM1 phosphatase activity does not contribute to the maintenance of most XLCNM phenotypes.

## Discussion

In this study, we investigated the involvement of MTM1 enzymatic activity on the phenotypes of XLCNM. Using heterologous expression of human genes in yeast, we showed that the PPIn phosphatase activity of MTM1 was directly linked to vacuolar homeostasis. The vacuolar phenotypes induced by expressing different MTM1 mutants found in patients and their measured impact on PPIn levels revealed that not all MTM1 mutants were associated to inactive phosphatase. In addition, using gene transfer in a murine model of XLCNM, we were able to significantly ameliorate most morphological phenotypes with two different phosphatase-inactive mutants of MTM1. Altogether, our data strongly suggest that the main roles of MTM1 in adult muscle are largely independent of its enzymatic activity, with the exception of triad shape and fiber size distribution.

We report here a sensitive assay to determine human MTM1 phosphatase activity in yeast *S. cerevisiae* using vacuole size as a read-out. We showed that vacuole size correlates with the levels of intracellular PtdIns3*P* and PtdIns(3,5)*P*_2_ dephosphorylated by MTM1. In yeast cells, a reverse correlation was observed between the *in vivo* phosphatase activity and the MTM1 protein level. Indeed, the enzymatically inactive MTM1^R421Q^ and MTM1^C375S^ are the most produced whereas the most active MTM1^R69C^ was the least abundant (Figure 1B). This suggests that yeast cells regulate the levels of human MTM1 to avoid massive deregulation of PPIn levels. This regulation could be post-transcriptional since the same effect was observed with two different replication origins (2 µ or CEN-ARS) combined with two different promoters, the yeast *PGK1* and the bacterial *tetO* promoters. Thus, an equilibrium between intracellular protein levels and PtdIns3*P* and PtdIns(3,5)*P*_2_ dephosphorylation rates may have been reached to ensure yeast growth in the presence of human MTM1 active forms. This is further supported by the fact that despite being massively produced in yeast, enzymatically active MTM1 did not drastically deplete intracellular PtdIns3*P* but restored similar PtdIns3*P* levels to the SEY6210 WT strain. It may also reflect a specificity of MTM1 towards distinct intracellular subpools of PtdIns3*P*.

**Figure 10:**
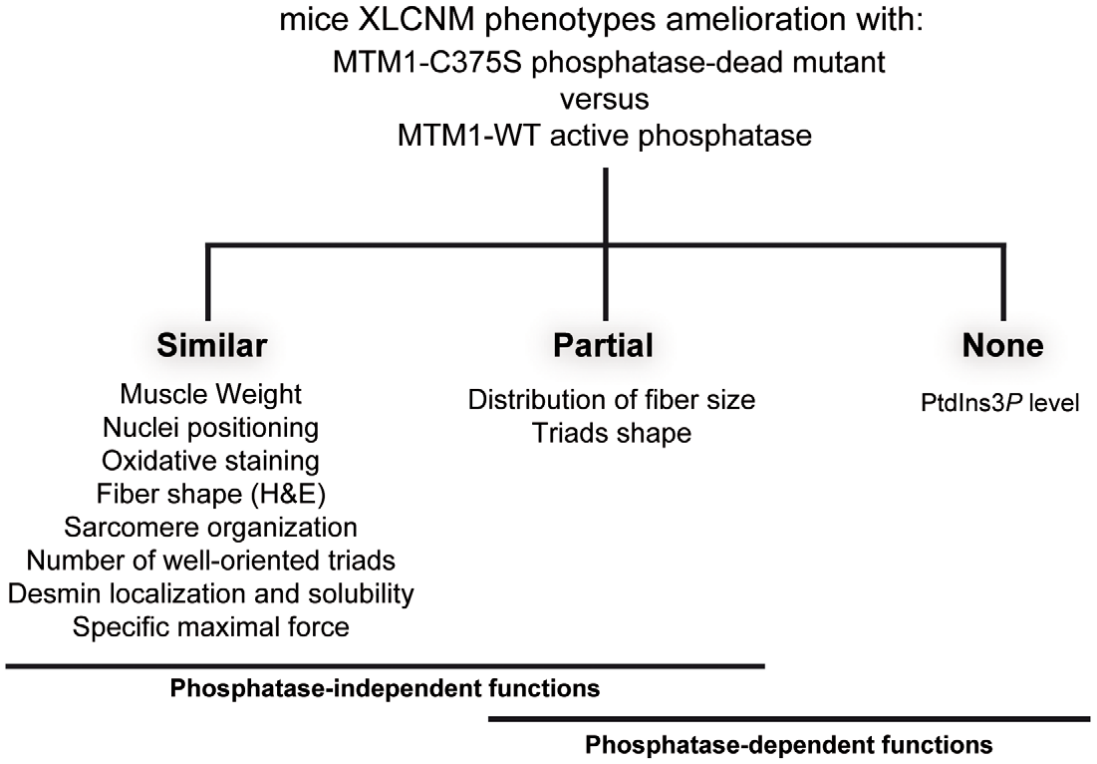
XLCNM phenotypes that are ameliorated by the MTM1-C375S phosphatase-dead mutant to a similar extend as with the wild-type MTM1, in the *Mtm1* KO muscle. Phosphate-independent and phosphatase-dependent functions are underlined.

Using different approaches in two eukaryotic models, the yeast *S. cerevisiae ymr1Δ* and the *Mtm1* KO mouse, our results indicate that the XLCNM disease is not solely linked to a defect in MTM1 phosphatase activity. In yeast cells, several XLCNM patient mutants responsible for severe forms of the disease displayed a phosphatase activity comparable to wild-type MTM1. In this model, the MTM1 phosphatase activity was linked to vacuolar homeostasis, in accordance with the known function of PtdIns3*P* and PtdIns(3,5)*P*_2_ in yeast cells [18]. In the *Mtm1* KO mice AAV gene transfer of wild-type MTM1 or phosphatase-inactive MTM1^C375S^ and MTM1^S376N^ mutants significantly improved the XLCNM phenotypes. Comparison of AAV-*Mtm1*-WT and AAV-*Mtm1*-CS injected *Mtm1* KO mice muscles revealed that ectopic expression of MTM1^C375S^ phosphatase-dead mutant corrected similarly as MTM1 wild-type: the muscle weight, nuclei positioning, oxidative staining and fiber shape (HE staining), desmin localization and solubility, sarcomere organization, the presence of well-oriented triads at the sarcomere and the specific maximal force, whereas the distribution of the fiber size and the triads shape were only partially ameliorated (Figure 10). Whether the complete correction of these phenotypes requires longer time of expression or the phosphatase activity of MTM1 remains an open question. Thus, apart from PtdIns3*P* levels, which are mainly dependent on the phosphatase activity, only fiber size distribution and triads shape appear both phosphatase-dependent and phosphate-independent functions of MTM1. Interestingly, since mice were injected with MTM1^C375S^ or MTM1^S376N^ at 3 weeks when animals start to present some pathological signs, it supports that these dead phosphatases did not only improve but were also able to revert the progression of the disease. Thus, even though the phosphatase activity of MTM1 is very important for its cellular function likely by impacting on vesicular trafficking, the loss of this activity is not responsible for the maintenance of most muscle phenotypes observed in the disease. This strongly suggests that defects in PPIn metabolism and vacuolar homeostasis are not the main cause in the maintenance of XLCNM phenotypes. However, we do not exclude that defect in the regulation of triad shape may affect muscle function at later stages, even so we did not observe significant differences in muscular specific maximal force after 4 weeks of transduction. Based on previous studies and on our results, we favor the hypothesis that the MTM1 phosphatase activity is crucial for the onset of the disease but less important for its maintenance in later stages of the myopathy. In this study only the PtdIns3*P* levels could be measured in the muscle, thus we cannot exclude that there might be a correlation with restored PtdIns(3,5)*P*_2_, although the MTM1^C375S^ mutant was shown to lack enzymatic activity against both PtdIns3*P* and PtdIns(3,5)*P*_2_ [28]. Moreover, Kiger and colleagues reported that down regulation of PI3K class II (Pi3K68D) in drosophila could rescue viability and several defects observed in mutant of mtm, the fly orthologue for MTM1, MTMR1 and MTMR2 [17], [38]. It is possible that in fly the role of myotubularins are more tightly linked to the phosphatase activity than in mammals where the diversification of myotubularins may have developed some phosphatase-independent and tissue-specific functions.

Mutations responsible for XLCNM are found all along the MTM1 protein [3], [6]. For most of them, including missense mutations, the MTM1 protein level was strongly decreased or not detectable in fibroblasts, lymphoblasts or myoblasts from patients, suggesting that XLCNM results in most cases from the absence or instability of MTM1 [8]. Recent results show that *Mtm1* p.R69C mice model is associated to mild CNM phenotypes with undetectable MTM1 protein levels, however the presence of residual protein that might account for the milder phenotype compared to the *Mtm1* KO mice cannot be ruled out [39]. One exception was the MTM1^S376N^ mutant associated to normal protein level in lymphoblast and leading to severe XLCNM [8], [9]. The MTM1^S376N^ mutant is phosphatase inactive *in vitro* [12] and *in vivo* (Figure S5). However, the level of this MTM1 mutant was not investigated in patients' muscle, as it requires a muscle biopsy from a patient deceased a long time ago in the neonatal period, and thus it is possible that this mutation leads to the instability of the protein in muscle. Moreover, Pierson et al. recently showed that the mutation predicted to lead to the R69C aminoacid change was in fact promoting a splicing defect and loss of the protein in the skeletal muscle of the R69C knock-in mice [39], suggesting that other missense mutations might impact on splicing in the diseased tissue. As exogenous expression of this MTM1^S376N^ mutant and of the phosphatase-dead MTM1^C375S^ improves muscle atrophy, fiber hypotrophy and organelles positioning defects, we conclude that these pathways are not mainly linked to the phosphatase activity but to other functions of MTM1. Based on these new findings, we rather propose that the MTM1 myotubularin protein might be a key effector involved in complex protein-protein interactions required for proper muscular functions. Indeed, MTM1 does not display a skeletal muscle-specific expression [40], whereas the XLCNM disease is mostly restricted to skeletal muscle. This would suggest that MTM1 interacts with muscle specific proteins and is required for their proper localization/function. Among these, the muscle-specific intermediate filament desmin involved in organelle positioning is a good candidate. MTM1 is required for proper desmin localization and assembly and some XLCNM-causing mutations disrupt the MTM1-desmin interaction [32]. Furthermore the MTM1^C375S^ phosphatase-dead mutant restores the abnormal desmin organization in MTM1-deficient muscle, suggesting that the phosphatase activity of MTM1 is not required for normal desmin organization and assembly in muscle fibers. Thus, the maintenance of desmin organization and IFs network is a phosphatase-independent function of MTM1 that is important for maintenance of the muscle structure and function.

Our work also shows that a disease due to mutation(s) affecting an enzyme is not always associated with loss of the corresponding enzymatic activity. Such dual function of PPIn metabolizing enzymes has been described for class I PI3K. Knock-out mice lacking PI3K protein expression show different phenotypes than knock-in mice expressing a kinase-dead mutant [41], [42]. Moreover, manipulation of myotubularins or other PPIn regulatory proteins by knock-down, overexpression or specific intracellular targeting is being widely used to decipher the roles of these lipids [43]. Our results call for cautiousness when interpreting the observed effects as they may result from a function unrelated to their enzymatic activity.

In conclusion, our data unravel an important and novel aspect of XLCNM as we provide evidences for a scaffolding activity of MTM1 for muscle specific proteins, such as desmin, which appears more important than its phosphatase activity in the maintenance of the XLCNM pathology. Our findings have important implications in the design of therapeutic approaches aiming to manipulate the phosphoinositide level in the different diseases linked to myotubularin homologues. Whether the MTM1 phosphatase activity is also dispensable for the development of the disease and the exact link between PPIn modulation and muscle function remains to be established.

## Materials and Methods

### Ethics statement

Animals were housed in a temperature-controlled room (19–22°C) with a 12:12-h light/dark cycle. Mice were humanely killed by CO2 inhalation followed by cervical dislocation, according to national and European legislations on animal experimentation.

### Plasmids, yeast strains, and media

The human *MTM1* ORF was cloned into pENTR™1A plasmid (Invitrogen) to generate an entry clone. Gateway system (Invitrogen) was used to clone the different *MTM1* constructs into the yeast expression vectors pVV200 and pVV204 or into a pAAV-MCS vector.

S. cerevisiae fab1Δ (MATα ura3Δ0, leu2Δ0, his3-Δ1, met15-Δ0, fab1::kanMX4) mutant (EUROSCARF collection), ymr1Δ (MATα ura3-52, leu2-3,112, his3-Δ200, trp1-Δ901, lys2-801, suc2-Δ9 ymr1::HIS3) and WT (SEY6210 strain; MATα ura3-52, leu2-3, 112, his3-Δ200, trp1-Δ901, lys2-801, suc2-Δ9) cells [21] were grown at 30°C in rich medium (YPD): 1% yeast extract, 2% peptone, 2% glucose or synthetic drop-out medium (SC): 0.67% yeast nitrogen base without amino acids, 2% glucose and the appropriate amino acids mixture to ensure plasmid maintenance.

### Western blot analysis

Yeast cells were lysed by glass beads using a FASTprep (MP Biomedicals) in PBS1X, sorbitol 0.3 M, Complete Mini EDTA-free protease inhibitor cocktail (Roche Diagnostics) and PMSF 1 mM. Lysates were cleared and analyzed by SDS-PAGE and Western blot using mouse monoclonal 1G6 anti-MTM1 (1/10,000) [8] and mouse monoclonal anti-PGK1 (1/400) (Invitrogen) antibodies. Muscles were homogenized in 50 mM Tris, 10% glycerol, 1 mM EDTA, 50 mM KCl, 10 mM beta-glycerophosphate, 10 mM NaF, 1 mM Na3VO4, 0.1% SDS, 2% Triton X-100 and protease inhibitors (Roche Diagnostics) using a Polytron homogenizer (Kinematica Inc.). Mouse anti-glyceraldehyde-3-phosphate dehydrogenase (Chemicon) and rabbit anti-MTM1 antibody (R2868) were used for detection.

### Yeast vacuolar staining

FM4-64 (Invitrogen) staining was performed as previously described [44]. Labeled yeast cells were observed by fluorescence microscopy (Axiovert200, Zeiss) in SC-trp medium. Cells were counted and classified into different categories: more than two lobes, small one or two lobes and unilobar large or giant vacuoles.

### Yeast phosphoinositide analysis

Labeling and lipid extraction procedures were done as previously described [45]. *ymr1Δ* cells expressing MTM1 were grown for 16 h in presence of 40 µCi/ml H_3_^32^PO_4_ (Perkin Elmer) before lysis by TCA. Lipids were extracted with 95% EtOH∶diethyl ether∶pyridine at 15∶5∶1 v/v. Samples were analyzed by TLC and labeled spots were identified by autoradiography and PPIn standards. Labeled PtdIns*P* as well as PtdIns*P*_2_ were scraped off the plates, collected and deacylated before being analyzed by high-performance liquid chromatography (HPLC) Whatman PartiSphere 5 SAX (4.6×125 mm) as previously described [46].

### PtdIns5*P* and PtdIns3*P* mass assay from yeast and muscle extracts

*ymr1Δ* cells producing the different MTM1 were grown to exponential phase, lipids extraction and TLC separation were performed as described above. Spots corresponding to PtdIns*P* were extracted and submitted to an *in vitro* kinase assay using recombinant PtdIns5*P* 4-kinase type IIα in presence of [γ-^32^P]-ATP [28], [29]. Among the different PtdIns*P* species, this kinase specifically phosphorylates PtdIns5*P* to PtdIns(4,5)*P*_2_ and the measured quantity of ^32^P-PtdIns(4,5)*P*_2_ will directly represent the *in vivo* PtdIns5*P* intracellular levels. The total lipids from TA muscles were extracted with the Dounce homogenizer using the method of Bligh and Dyer [47] and prepared for mass assay to measure the intracellular PtdnIs3*P* levels in muscle by a novel mass assay using recombinant PIKfyve kinase in presence of [γ-^32^P]-ATP [35].

### Production and purification of Adeno-Associated Virus (rAAV)

rAAV2/1 vectors were generated by a triple transfection of AAV-293 cell line with pAAV2-insert containing the insert under the control of the CMV promoter and flanked by serotype-2 inverted terminal repeats, pXR1 containing rep and cap genes of AAV serotype-1, and pHelper encoding the adenovirus helper functions. Viral vectors were purified and quantified by real time PCR using a plasmid standard pAAV-eGFP. Titers are expressed as viral genomes per ml (vg/ml) and rAAV titers used here were 5–7.10^11^ vg/ml.

### AAV–transduction of wild-type tibialis anterior muscle of mice

Two to three week-old male wild-type and *Mtm1* KO 129PAS mice were anesthetized by intraperitoneal injection of 5 µl/body gram of ketamine (20 mg/ml, Virbac) and xylazine (0.4%, Rompun, Bayer). Tibialis anterior (TA) muscles were injected with 20 µl of AAV2/1 preparations, or AAV2/1 empty virus solution. Animals were housed in a temperature-controlled room (19–22°C) with a 12:12-h light/dark cycle. Mice were humanely killed by CO_2_ inhalation followed by cervical dislocation, according to national and European legislations on animal experimentation. TA muscles were dissected 4 weeks after injection and frozen in nitrogen-cooled isopentane and liquid nitrogen for histological and immunoblot assays, respectively.

### Functional analysis of the muscle

Muscle force measurements were evaluated by measuring *in situ* muscle contraction in response to nerve and muscle stimulation, as described previously. Animals were anesthetized by intraperitoneal injection of pentobarbital sodium (50 mg per kg). The distal tendon of the TA was detached and tied with a silk ligature to an isometric transducer (Harvard Bioscience, Holliston, MA). The sciatic nerve was distally stimulated, response to tetanic stimulation (pulse frequency of 50 to 143 Hz) was recorded, and absolute maximal force was determined. After contractile measurements, the animals were sacrificed by cervical dislocation. To determine specific maximal force, TA muscles were dissected and weighted.

### Histological and immunofluorescence analysis of skeletal muscle

Transverse cryosections (8 µm) of mouse TA skeletal muscles were stained with hematoxylin and eosin (HE), succinate dehydrogenase (SDH) and viewed with a fluorescence microscope (DM4000; Leica Microsystems, Sunnyvale, CA). Cross-sectional area (CSA) was analyzed on HE sections from TA mouse skeletal muscles, using the RoiManager plugin of ImageJ image analysis software (Rasband, W.S., ImageJ, U. S. National Institutes of Health, Bethesda, Maryland, USA, http://rsb.info.nih.gov/ij/, 1997–2009). The percentage of TA muscle fibres with centralized or internalized nuclei was counted using the cell counter plugin of ImageJ image analysis software. Transverse cryosections (8 µm) sections of mouse TA skeletal muscles were prepared, fixed, and stained with antibodies to desmin (Santa Cruz). Nuclei were detected by co-staining with Hoechst (Sigma-Aldrich) for 10 minutes. Sections were viewed using a fluorescence microscope (DM4000; Leica Microsystems, Sunnyvale, CA).

### Transmission electron microscopy

Muscle biopsies from TA muscles of anesthetized mice were fixed with 4% PFA and 2.5% glutaraldehyde in 0.1 M phosphate buffer (pH 7.2) and processed as described [30]. Determination of the triads organization was accomplished on images at the magnification of ×25,000. The triad structure was identified using morphological criteria on the longitudinal sections of the muscle and the number of triads per sarcomere was quantified. Ratio of triads/sarcomere was calculated by dividing number of triad structure identified by the total number of sarcomere present on the section.

### Microsome preparations

Frozen muscles were homogenized to prepare membranous (microsomal) fractions from skeletal muscles [32]. Membrane fractionation was confirmed using several protein markers: mouse anti-SERCA1 (MA3–911; ABR) and mouse anti-α-sarcoglycan [48], cytoplasmic protein mouse anti-β-tubulin (IGBMC antibody facility) and nucleus protein mouse anti-TATA-box-binding protein (IGBMC antibody facility).

### Desmin solubility assays

Cells or muscles were treated as described in [32] with the following modifications. Extracts were obtained by homogenization in extraction buffer (50 mM Tris-Cl pH 7.5, 50 mM NaCl, 5 mM EDTA, 5 mM EGTA, 1 mM DTT, 0,5% Triton X-100, 2 mM PMSF) supplemented with complete protease inhibitor tablet (Roche), 1 mM Leupeptin and 1 mM pepstatin A (SIGMA). Equal weight of tibialis anterior muscles were homogenized with a Polytron homogenizer (Kinematica Inc.) in ice-cold extraction buffer supplemented with 0.05% (w/v) SDS. The muscle extracts were incubated ON at 4°C in the extraction buffer with 0.1% of N-Lauroylsarcosine Sodium Salt solution (SIGMA). Muscle extracts were centrifuged during 30 min at 30,000 rpm at 4°C. Pellets were collected as the insoluble material and solubilized in extraction buffer supplemented with 8 M Urea.

Extended experimental procedures are available in Text S1.

## Supporting Information

Figure S1 Analysis of growth upon MTM1 expression in *ymr1Δ* yeast cells. (A) Drop test growth assays on *ymr1Δ* mutant yeast cells transformed or not with pVV204 (CEN, expression) or pVV200 (2 µ, overexpression) plasmids bearing the different MTM1 proteins. Mid-log phase cultures of the indicated yeast cells were serially diluted to the indicated OD_600_ and spotted onto YPD medium. Growth was evaluated after 2 days of incubation at 30°C. (B) Growth curves of *ymr1Δ* cells bearing pVV200 (2 µ) plasmid either empty or coding for different MTM1 forms. Cell concentrations were measured by OD_600 nm_ at the indicated time after incubation at 30°C. The growth curve corresponds to the logarithmic curve.
(TIF)

Figure S2 Analysis of vacuolar morphology upon MTM1 expression in wild-type yeast cells. (A) Anti-MTM1 Western-blot on wild-type yeast protein extracts. Protein extracts of wild-type yeast cells transformed or not with pVV204 (CEN, expression) or pVV200 (2mu, overexpression) empty plasmids or bearing the indicated MTM1 constructs were analyzed by Western-blot with the monoclonal 1G6 anti-MTM1 antibody. Degradation products could be seen associated to high expression. (B) Mid-log phase yeast cells cultures of wild-type (WT) yeast strain transformed or not with pVV204 (CEN) or pVV200 (2 µ) plasmids bearing the different MTM1 forms were serially diluted to the indicated OD_600_ and spotted on YPD plates. Growth was evaluated after 2 days of incubation at 30°C. (C) Quantification of the different vacuolar morphologies observed in wild-type yeast cells (SEY6210 WT) producing MTM1, MTM1^C375S^, MTM1^V49F^, MTM1^R69C^, MTM1^N180K^ or MTM1^R421Q^ on either pVV204 (CEN, expression) or pVV200 (2 µ, overexpression) plasmid. For each strain, 300 to 600 cells were observed by microscopy (DIC and FM4-64) and sorted into one of the three categories: unilobar large or giant (in white), small one or two lobes (in grey) and more than two lobes or fragmented (in black) vacuoles. A scheme representing these three classes of vacuolar phenotypes is presented at the top of the graph. Histograms show the proportion of each class in the different transformed yeast cells.
(TIF)

Figure S3 Subcellular distribution of the different MTM1 mutants. Total yeast protein extracts of *ymr1Δ* cells expressing wild-type MTM1 or the different mutants from pVV204 (CEN) were subjected to differential centrifugation. P13 and P100 pellet fractions represent the high-density membrane fractions, and the supernatant S100 the soluble fraction. Equivalent amounts of proteins were loaded, separated by SDS-PAGE and analyzed by western-blot. MTM1 was detected with mouse monoclonal 1G6 antibodies. The transmembrane sorting receptor Vps10 and the cytosolic 3-phosphoglycerate kinase Pgk1 were used as markers.
(TIF)

Figure S4 In vitro phosphatase activity assays on the different MTM1 mutants. Yeast protein extracts from *ymr1Δ* cells transformed with pVV200 (overexpression) plasmid either empty or coding for wild type MTM1 protein or the different mutants were subjected to anti-MTM1 immunoprecipitation. After control by anti-MTM1 Western-blot, comparable amounts of MTM1 were tested for *in vitro* phosphatase activity using fluorescent C6-BODIPY-FL-PPIn, according to [49] and [28]. (A) The products of the enzymatic reaction were separated by TLC (Thin Layer Chromatography) allowing the different PPIn species (PtdIns, PtdIns monophosphate and PtdIns bisphosphate) to migrate at a different height. Fluorescent PtdIns, PtdIns3*P*, PtdIns5*P* (barely detectable) and PtdIns(3,5)*P*_2_ were spotted on the TLC and used as controls for the TLC migration (phosphoinositides). The TLC plate was revealed under a UV table. (B) Percentage of hydrolysis of the fluorescent phosphoinositide substrates (PtdIns3*P* in blue and PtdIns(3,5)*P*_2_ in red) are reported for each construct.
(TIF)

Figure S5 Model of MTM1-C375S and MTM1-S376N. The crystal structure of MTMR2 (PDB accession number 1ZSQ) was used to model the MTM1 catalytic pocket that shares the same amino acids. The figure was prepared with the PyMOL software (The PyMOL Molecular Graphics System, Version 1.5.0.1 Schrödinger, LLC.). (A) MTM1-C375S model. (B) MTM1-S376N model. (C) The MTM1-S376N mutant is catalytically inactive *in vivo* in yeast cells. Protein extracts of *ymr1Δ* cells transformed with pVV204 (CEN) or pVV200 (2 µ) plasmids bearing the MTM1-S376N mutant were analyzed by Western-blot. MTM1 production was detected with the mouse monoclonal 1G6 anti-MTM1 antibody. Protein loading was evaluated by immunodetection of the yeast endogenous 3-phosphoglycerate kinase Pgk1 protein. (D) Quantification of the different vacuolar morphologies observed in *ymr1Δ* yeast cells producing MTM1, MTM1^C375S^ or MTM1^S376N^ on pVV200 (2 µ, overexpression) plasmid. For each strain, 300 to 500 cells were observed by microscopy (DIC and FM4-64) and sorted into one of the three categories: unilobar large or giant (in white), small one or two lobes (in grey) and more than two lobes or fragmented (in black) vacuoles. Histograms show the proportion of each class in the different transformed yeast cells. (E) Quantitative analysis of PtdIns5*P* produced in *ymr1Δ* cells transformed with the pVV204 (CEN, expression) plasmid empty or bearing MTM1 wild-type, MTM1-C375S or MTM1-S376N mutant. The intracellular levels of PtdIns5*P* are expressed as pmol for 200 units of OD_600 nm_ of yeast cells. The graphs represent the mean of two independent experiments shown with the standard deviation.
(TIF)

Text S1 Extended experimental procedures.
(PDF)
