# Supplementary figures and images for: Phosphatase-Dead Myotubularin Ameliorates X-Linked Centronuclear Myopathy Phenotypes in Mice
Source: PLoS Genet. 2012 Oct 11;8(10):e1002965. doi: 10.1371/journal.pgen.1002965 (PMC3469422; doi:10.1371/journal.pgen.1002965)

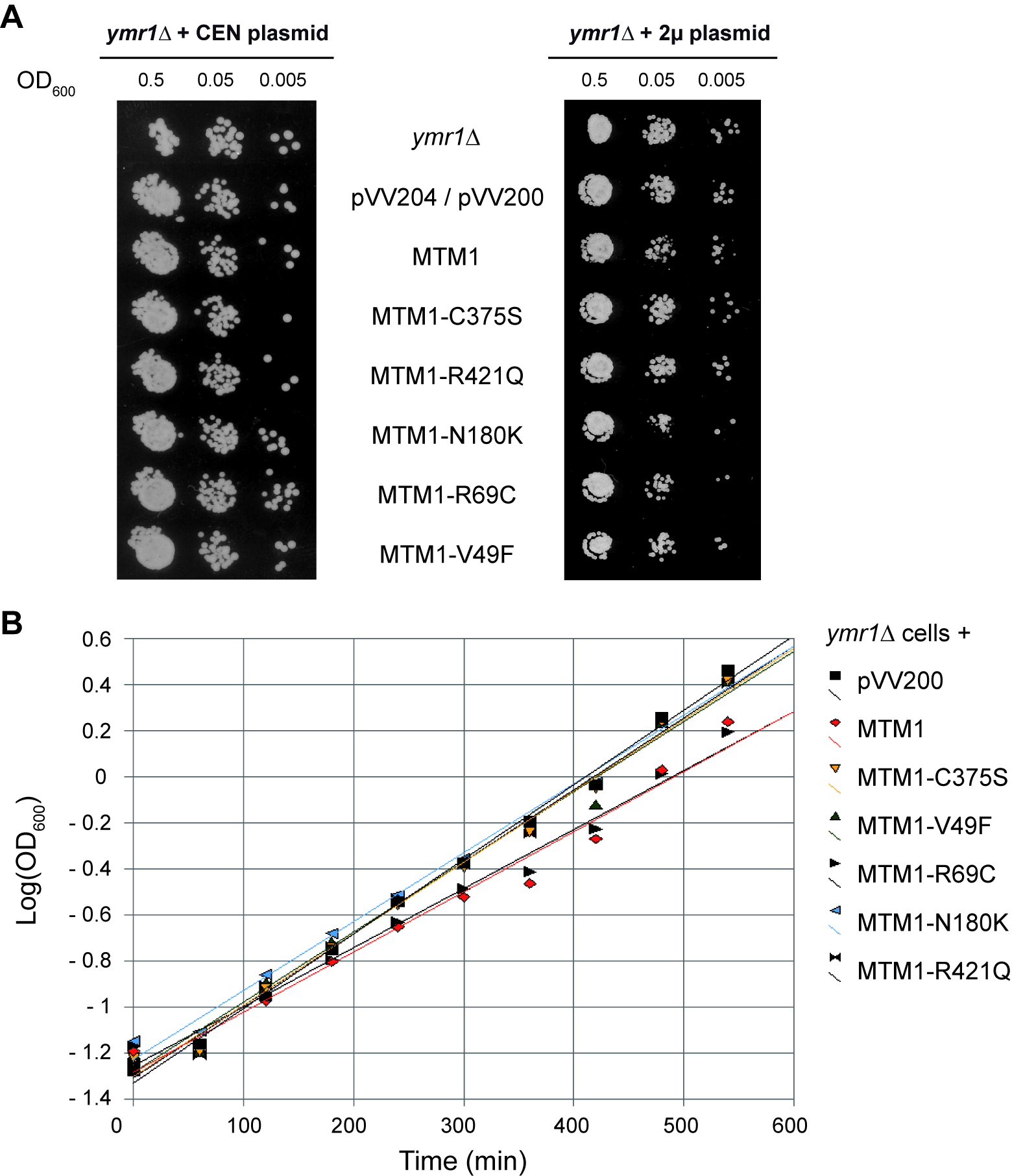

Supplement: Figure S1 — Analysis of growth upon MTM1 expression in ymr1Δ yeast cells. (A) Drop test growth assays on ymr1Δ mutant yeast cells transformed or not with pVV204 (CEN, expression) or pVV200 (2 µ, overexpression) plasmids bearing the different MTM1 proteins. Mid-log phase cultures of the indicated yeast cells were serially diluted to the indicated OD600 and spotted onto YPD medium. Growth was evaluated after 2 days of incubation at 30°C. (B) Growth curves of ymr1Δ cells bearing pVV200 (2 µ) plasmid either empty or coding for different MTM1 forms. Cell concentrations were measured by OD600 nm at the indicated time after incubation at 30°C. The growth curve corresponds to the logarithmic curve. (TIF) [file pgen.1002965.s001.tif]

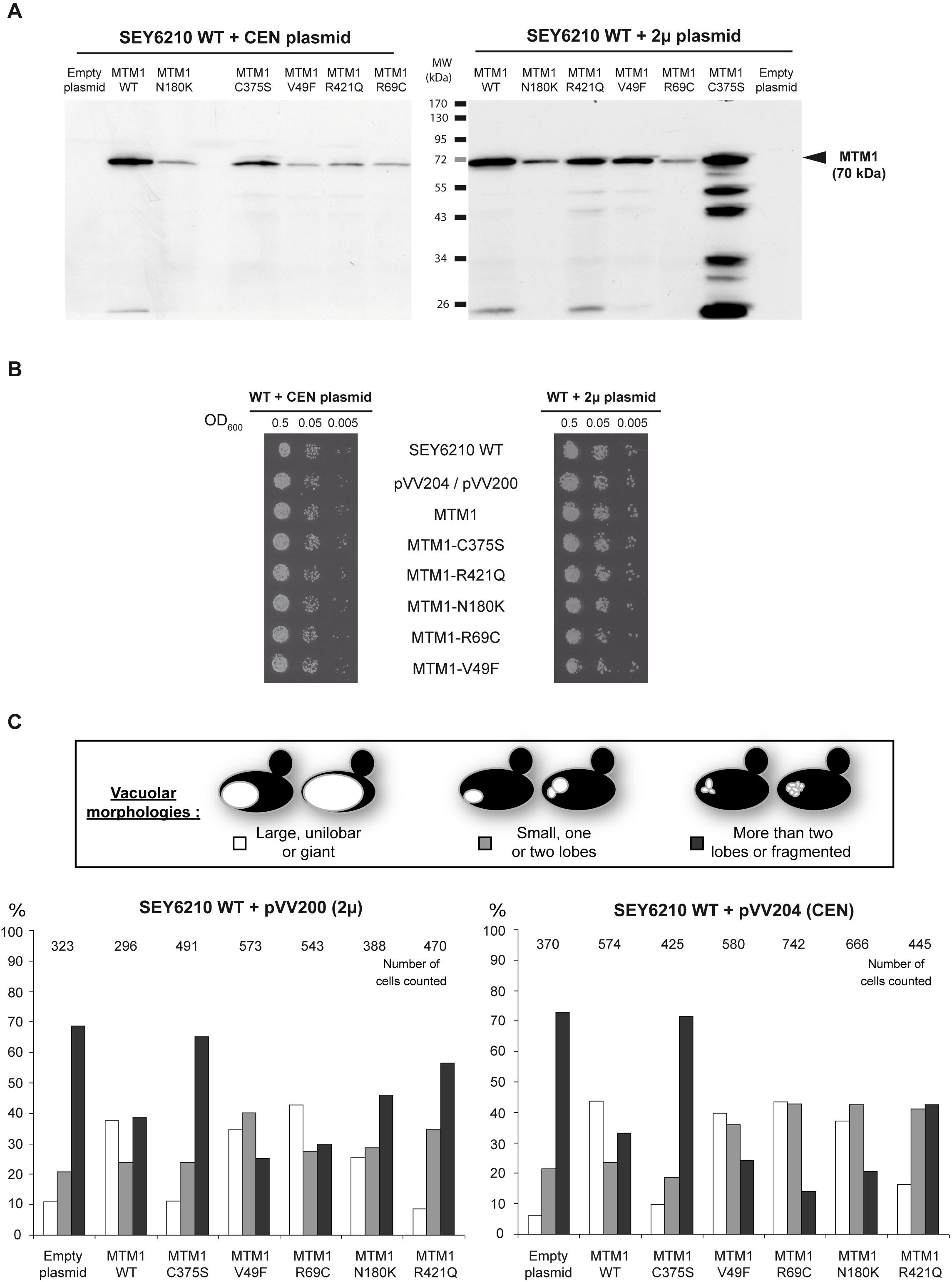

Supplement: Figure S2 — Analysis of vacuolar morphology upon MTM1 expression in wild-type yeast cells. (A) Anti-MTM1 Western-blot on wild-type yeast protein extracts. Protein extracts of wild-type yeast cells transformed or not with pVV204 (CEN, expression) or pVV200 (2mu, overexpression) empty plasmids or bearing the indicated MTM1 constructs were analyzed by Western-blot with the monoclonal 1G6 anti-MTM1 antibody. Degradation products could be seen associated to high expression. (B) Mid-log phase yeast cells cultures of wild-type (WT) yeast strain transformed or not with pVV204 (CEN) or pVV200 (2 µ) plasmids bearing the different MTM1 forms were serially diluted to the indicated OD600 and spotted on YPD plates. Growth was evaluated after 2 days of incubation at 30°C. (C) Quantification of the different vacuolar morphologies observed in wild-type yeast cells (SEY6210 WT) producing MTM1, MTM1C375S, MTM1V49F, MTM1R69C, MTM1N180K or MTM1R421Q on either pVV204 (CEN, expression) or pVV200 (2 µ, overexpression) plasmid. For each strain, 300 to 600 cells were observed by microscopy (DIC and FM4-64) and sorted into one of the three categories: unilobar large or giant (in white), small one or two lobes (in grey) and more than two lobes or fragmented (in black) vacuoles. A scheme representing these three classes of vacuolar phenotypes is presented at the top of the graph. Histograms show the proportion of each class in the different transformed yeast cells. (TIF) [file pgen.1002965.s002.tif]

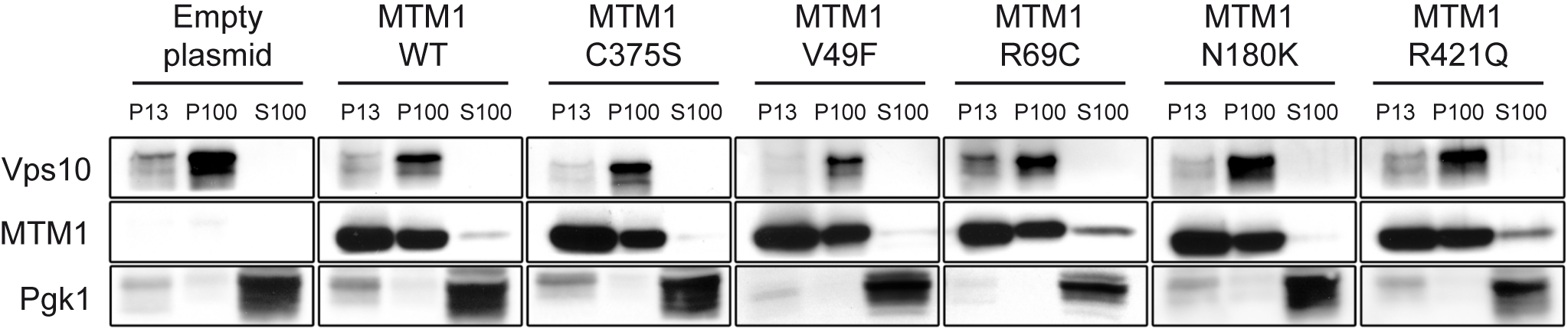

Supplement: Figure S3 — Subcellular distribution of the different MTM1 mutants. Total yeast protein extracts of ymr1Δ cells expressing wild-type MTM1 or the different mutants from pVV204 (CEN) were subjected to differential centrifugation. P13 and P100 pellet fractions represent the high-density membrane fractions, and the supernatant S100 the soluble fraction. Equivalent amounts of proteins were loaded, separated by SDS-PAGE and analyzed by western-blot. MTM1 was detected with mouse monoclonal 1G6 antibodies. The transmembrane sorting receptor Vps10 and the cytosolic 3-phosphoglycerate kinase Pgk1 were used as markers. (TIF) [file pgen.1002965.s003.tif]

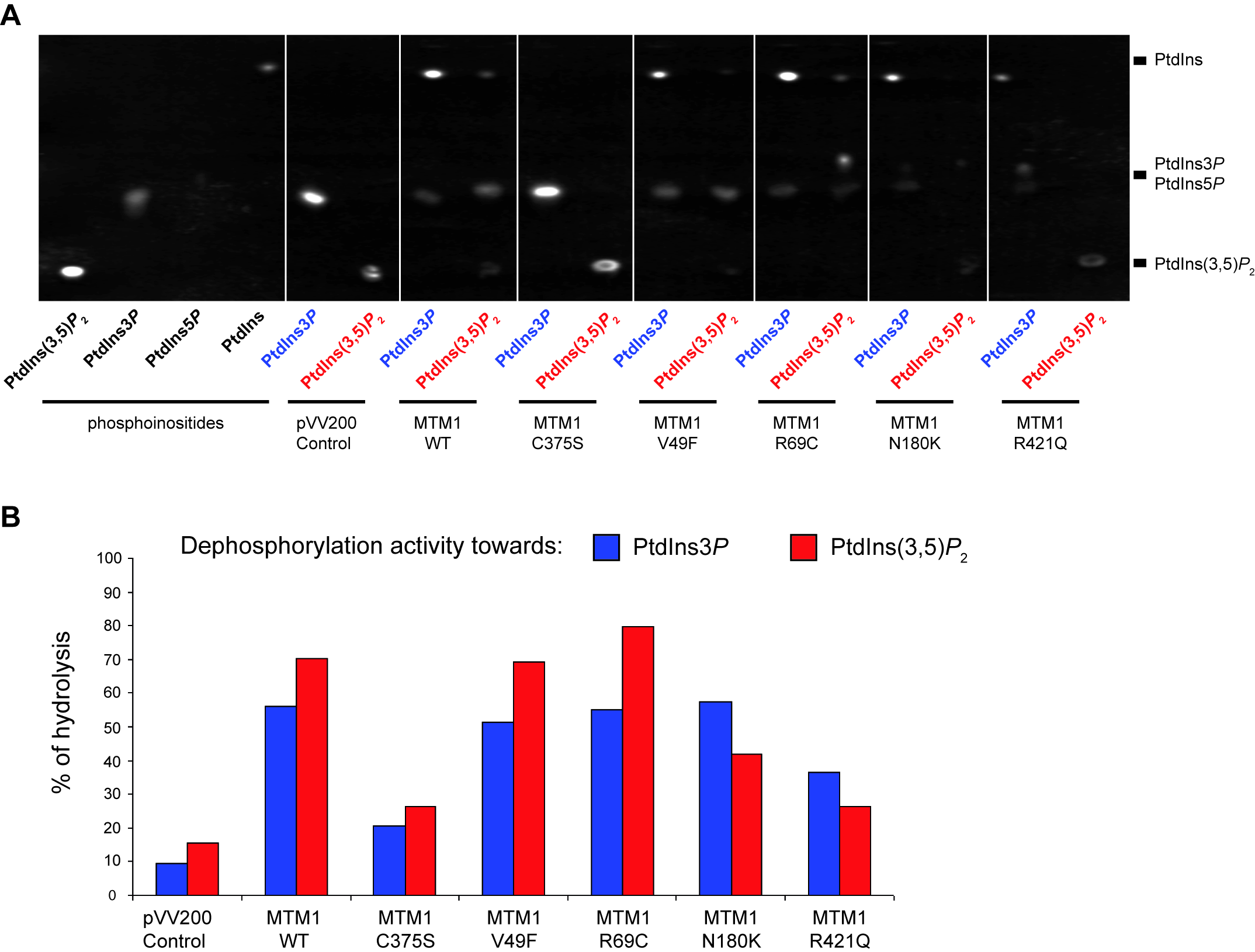

Supplement: Figure S4 — In vitro phosphatase activity assays on the different MTM1 mutants. Yeast protein extracts from ymr1Δ cells transformed with pVV200 (overexpression) plasmid either empty or coding for wild type MTM1 protein or the different mutants were subjected to anti-MTM1 immunoprecipitation. After control by anti-MTM1 Western-blot, comparable amounts of MTM1 were tested for in vitro phosphatase activity using fluorescent C6-BODIPY-FL-PPIn, according to [49] and [28]. (A) The products of the enzymatic reaction were separated by TLC (Thin Layer Chromatography) allowing the different PPIn species (PtdIns, PtdIns monophosphate and PtdIns bisphosphate) to migrate at a different height. Fluorescent PtdIns, PtdIns3P, PtdIns5P (barely detectable) and PtdIns(3,5)P 2 were spotted on the TLC and used as controls for the TLC migration (phosphoinositides). The TLC plate was revealed under a UV table. (B) Percentage of hydrolysis of the fluorescent phosphoinositide substrates (PtdIns3P in blue and PtdIns(3,5)P 2 in red) are reported for each construct. (TIF) [file pgen.1002965.s004.tif]

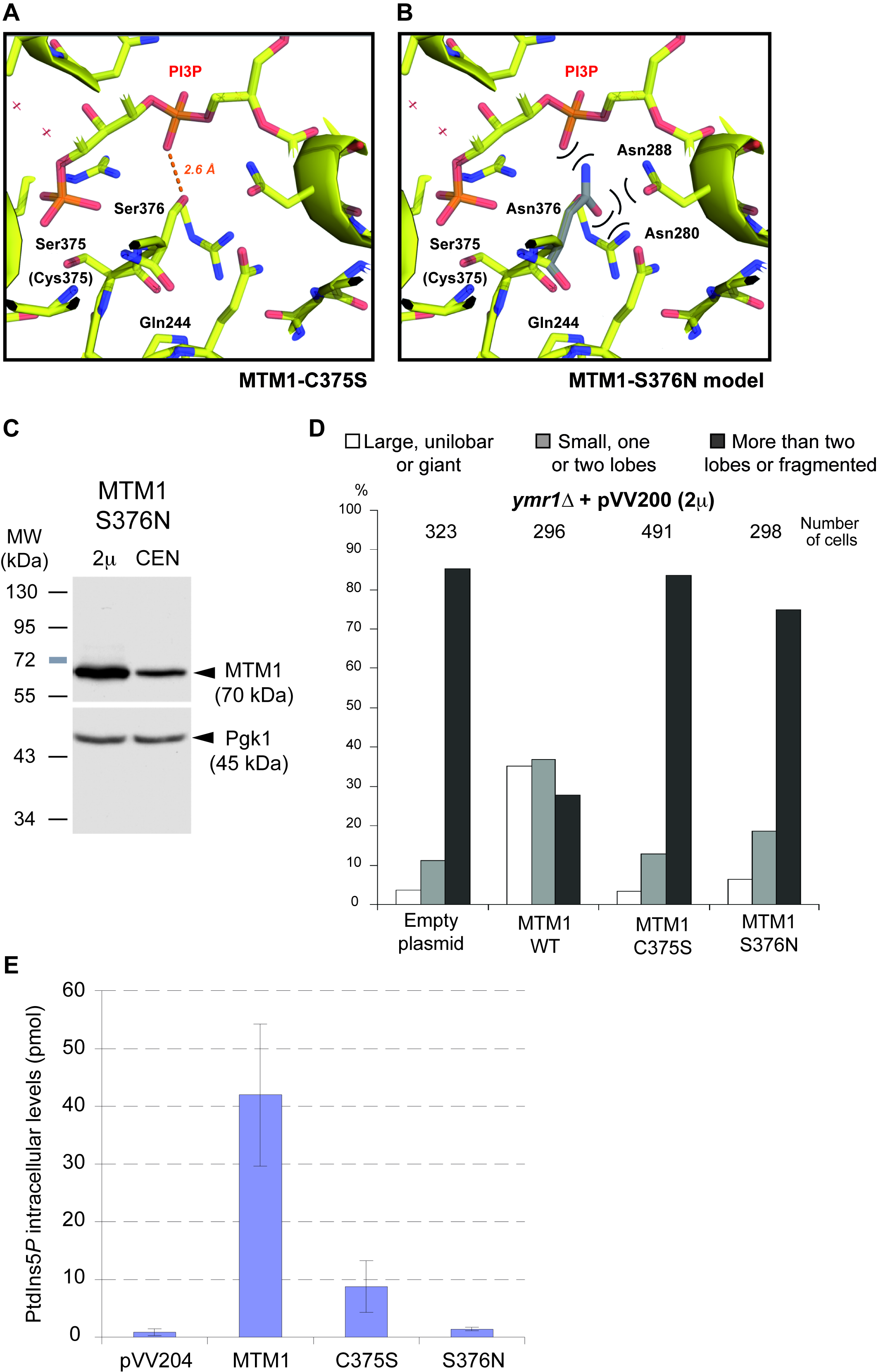

Supplement: Figure S5 — Model of MTM1-C375S and MTM1-S376N. The crystal structure of MTMR2 (PDB accession number 1ZSQ) was used to model the MTM1 catalytic pocket that shares the same amino acids. The figure was prepared with the PyMOL software (The PyMOL Molecular Graphics System, Version 1.5.0.1 Schrödinger, LLC.). (A) MTM1-C375S model. (B) MTM1-S376N model. (C) The MTM1-S376N mutant is catalytically inactive in vivo in yeast cells. Protein extracts of ymr1Δ cells transformed with pVV204 (CEN) or pVV200 (2 µ) plasmids bearing the MTM1-S376N mutant were analyzed by Western-blot. MTM1 production was detected with the mouse monoclonal 1G6 anti-MTM1 antibody. Protein loading was evaluated by immunodetection of the yeast endogenous 3-phosphoglycerate kinase Pgk1 protein. (D) Quantification of the different vacuolar morphologies observed in ymr1Δ yeast cells producing MTM1, MTM1C375S or MTM1S376N on pVV200 (2 µ, overexpression) plasmid. For each strain, 300 to 500 cells were observed by microscopy (DIC and FM4-64) and sorted into one of the three categories: unilobar large or giant (in white), small one or two lobes (in grey) and more than two lobes or fragmented (in black) vacuoles. Histograms show the proportion of each class in the different transformed yeast cells. (E) Quantitative analysis of PtdIns5P produced in ymr1Δ cells transformed with the pVV204 (CEN, expression) plasmid empty or bearing MTM1 wild-type, MTM1-C375S or MTM1-S376N mutant. The intracellular levels of PtdIns5P are expressed as pmol for 200 units of OD600 nm of yeast cells. The graphs represent the mean of two independent experiments shown with the standard deviation. (TIF) [file pgen.1002965.s005.tif]
