# Supplementary material for: Phosphatase-Dead Myotubularin Ameliorates X-Linked Centronuclear Myopathy Phenotypes in Mice
Source: PLoS Genet. 2012 Oct 11;8(10):e1002965. doi: 10.1371/journal.pgen.1002965 (PMC3469422; doi:10.1371/journal.pgen.1002965)
Supplement: Text S1 — Extended experimental procedures. (PDF) [file pgen.1002965.s006.pdf]

## Supplementary materials and methods

### Plasmids, Yeast Strains and media

The human *MTM1* ORF was cloned into the BamHI and EcoRV sites of pENTR™ 1A plasmid (Invitrogen) to generate an entry clone bearing *MTM1* ORF flanked by attL1 and attL2 sites. The different *MTM1* mutants were created by mutagenesis on the pENTR1A-MTM1 vector using the QuikChange® site-directed mutagenesis kit (Stratagene). Gateway system LR reactions (Invitrogen) were performed to clone the different *MTM1* constructs into the yeast destination expression vectors pVV200 and pVV204 (obtained from the European Saccharomyces cerevisiae Archive for Functional Analysis EUROSCARF) [3] or into a pAAV-MCS vector. All constructs were verified by sequencing. pXR1 (AAV1 serotype) plasmid was a kind gift from Jude Samulski at the Gene Therapy Center, The University of North Carolina at Chapel Hill, USA. The AAV Helper-Free system was purchased from Stratagene (La Jolla, CA, Catalog number 240071).

*S. cerevisiae* WT SEY6210 (*MATα ura3-52, leu2-3, 112, his3-Δ200, trp1-Δ901, his2-801, suc2-Δ9*) and *ymr1Δ* (*MATα ura3-52, leu2-3, 112, his3-Δ200, trp1-Δ901, his2-801, suc2-Δ9 ymr1::HIS3*) strains were a kind gift from Scott Emr [4]. Yeast cells were transformed using lithium acetate [5]. Transformants bearing the pVV200 and pVV204 derived plasmids were selected on SC-Trp medium.

### Western Blot analysis and subcellular fractionation

Muscles were homogenized in 50 mM Tris, 10% glycerol, 1 mM EDTA, 50 mM KCl, 10 mM beta-glycerophosphate, 10 mM NaF, 1 mM Na3VO4, 0.1% SDS, 2% Triton X-100 and protease inhibitors (PIC, Roche Diagnostics) using a Polytron® homogenizer (Kinematica Inc.), kept on ice for 10 min, and centrifuged at 2,000 rpm (5417R, Eppendorf) for 5 min to collect the supernatant and proteins were quantified with the Biorad Protein Assay detection kit. Proteins were transferred to nitrocellulose membranes and revealed by ECL chemiluminescence after incubation with primary then HRP-coupled secondary antibodies. Mouse anti-glyceraldehyde-3-phosphate dehydrogenase was from Chemicon (Temecula, CA; GAPDH, MAB374). Rabbit anti-MTM1 antibody (R2868) was made at the IGBMC polyclonal antibody facility against the murine sequence TSSSSQMVPHVQTHF. Secondary antibodies against mouse and rabbit IgG, conjugated with horseradish peroxidase (HRP) were obtained from Jackson ImmunoResearch

Laboratories (West Grove, PA). ECL chemiluminescent reaction kit was purchased from Pierce.

Yeast *ymr1Δ* transformed cells were grown in SC-Trp medium until $OD_{600nm}$=0.5-0.8. Cells were harvested by a 1,600x*g* centrifugation for 5 min at 4°C and washed once in PBS1X, sorbitol 0.3 M. Pellets were resuspended in 1 mL lysis buffer (PBS1X, sorbitol 0.3 M, Complete Mini EDTA-free protease inhibitor cocktail™ (Roche Diagnostics), PMSF 1 mM and after addition of 1 mL of 0.45 mm glass beads, cells were broken at 4°C using a FASTprep® (MP Biomedicals) with 5 runs of 30 sec under strong agitation (6.5 M/S) and cooled on ice for 1 min between each run. Lysates were cleared by a 5 min centrifugation at 800x*g*, supernatants representing total yeast protein extracts were collected, and protein concentrations were determined by using the Biorad Protein Assay detection kit.

Yeast subcellular fractionation was performed by differential centrifugation on the total protein extracts as previously described [6]. The lysate was centrifuged at 13,000x*g* for 10 min at 4°C. The pellet (P13) was kept for analysis and the supernatant was subjected to centrifugation at 100,000x*g* for 1h at 4°C. The resulting pellet (P100) and supernatant (S100) were collected and S100 represents the cytoplasmic fraction. All pellets (P13 and P100) representing the membrane fractions were resuspended in 200 $\mu$l of cytosol buffer (20mM Hepes pH6.8, 0.15M KoAc, 10mM $MgCl_2$, 0.25M Sorbitol). All fractions were analyzed by SDS-PAGE and Western blot using ECL protocols (GE Healthcare). Mouse monoclonal 1G6 anti-MTM1 (1/10,000) [7], mouse monoclonal anti-PGK1 (1/400) (Invitrogen), anti-Vps10 (1/200) (Invitrogen) and HRP-coupled anti-mouse Ig (1/10,000) (GE Healthcare) antibodies were used.

**FM4-64 staining and vacuolar morphologies quantification**

N-[3-triethylammoniumpropyl]-4-[ρ-diethylaminophenylhexatrienyl] pyridinium dibromide (FM4-64, Invitrogen) staining was performed by incubating YPD resuspended yeast cells with 16 μM of FM4-64 for 15 min at 25°C [8]. Labeled yeast cells were then harvested and resuspended in 1 mL fresh selective medium for a 1h chase at 25°C, then washed once in selective medium. Cells were then observed in the selective medium using fluorescence microscopy (Axiovert200, Zeiss, 100X objective, DIC and TRITC filters). Images were acquired with the Axiovision (Zeiss) software using the CoolSnapHQ2 camera (Roper Scientific) and processed with the ImageJ software (Rasband, W.S., ImageJ, U. S. National Institutes of Health, Bethesda, Maryland, USA,

http://imagej.nih.gov/ij/, 1997-2011). For each strain, at least 300 cells were counted and classified into one of the three categories: unilobar large or giant, small one or two lobes and more than two lobes or fragmented vacuoles. The small or large unilobar vacuoles classes correspond respectively to a unique vacuole filling either less than 50% of the cell volume (in grey), or more than 50% of the cell volume (in white).

**Immunoprecipitation**

For one immunoprecipitation assay, 30 $\mu$L protein A-Sepharose, 30 $\mu$L protein G-Sepharose and 30 $\mu$L γ-bind-Sepharose beads (GE Healthcare) were mixed, before to be washed twice in prechilled PBS1X and coated under gentle agitation on a wheel at 4°C for 90 min with 500 $\mu$L of PBS 1X, BSA 2%, NP40 0.5% buffer and 5 $\mu$L of mouse monoclonal anti-MTM1 1G6 antibodies. 5 mg of total yeast protein extract was incubated with the beads mix for 15 h at 4°C on a wheel. Beads were then washed 5 times in cold PBS1X + proteases inhibitors. The beads pellets were resuspended in 20 $\mu$L MTM1 phosphatase reaction buffer (Ammonium acetate 1 M + dithiothreitol pH6 0,2 mM) [1].

***In vitro* phosphoinositide phosphatase assay**

Phosphatase reaction assays were performed using 0.6 $\mu$g fluorescent phosphoinositide (BODIPY®-FL PPIn, C6, Echelon) and 10 to 20 $\mu$L Sepharose beads [2]. Phosphatase assays were incubated for 30 min at 37°C in 45 $\mu$L of MTM1 phosphatase reaction buffer. Reactions were terminated by addition of 100 $\mu$l of cold acetone and then evaporated in a Speed-Vac evaporator set on low heat [1,2]. The dried phosphatase assay products were resuspended in 6 $\mu$l of methanol and spotted onto a pretreated (methanol/water (3/2) + potassium oxalate 1%) glass-backed TLC plate (Merck TLC Silica Gel60 F254 20x20cm) and were allowed to migrate. The TLC plate was developed in a solvent system consisting of dichloromethane, MetOH, water, concentrated ammonium hydroxide (Roth, Rotipuran® Supra, 20%) (90/70/17/3) and then air-dried. Fluorescent lipids were visualized by UV light using a transilluminator (Appligen, Oncor).

**Yeast phosphoinositide measurements by metabolic labeling**

Labeling and lipid extraction procedures were done as previously described [9]. *ymr1Δ* yeast cells producing the different forms of MTM1 were grown in 5 mL of selective medium containing 40 μCi/ml $H_3{}^{32}PO_4$ (NEX054, Perkin Elmer) during 16h at 30°C. At

$OD_{600nm}$ 0.8-1 cells were lysed by the addition of TCA at a final concentration of 5% and incubated 1h on ice. After a 10,000x*g* centrifugation for 3 min at 4°C, pellets were washed once in chilled water and resuspended in 200μL water. 300μL of EEP extraction solution (95% EtOH :diethyl ether :pyridine at 15 :5 :1 v/v) was added. Lipids were extracted at 57°C for 30 minutes under shaking. Undissolved materials were pelleted by a 5 minutes centrifugation at 10,000x*g* and supernatants were collected in glass tubes for evaporation under argon flow. Dried lipids were dissolved in 100 μL chloforme/methanol (10:10) and evaporated under argon until 10-20 μL remained in the tubes. Samples were spotted on oxalate pre-treated TLC plates (MERCK TLC Silica Gel60 $F_{254}$ 20x20cm). Migration was performed with chloroform/methanol/$NH_4OH$ 10% (9 :7 :2 v/v) in a hermetic chamber until the solvent migration front reached 2 cm from the top. Labeled spots were identified by autoradiography and PPIn standards. Labeled PtdIns*P* as well as PtdIns$P_2$ were scraped off the plates, collected and deacylated before being analyzed by high-performance liquid chromatography (HPLC) Whatman PartiSphere 5 SAX (4.6 x 125 mm) as previously described [10].

**PtdIns5*P* mass assay in yeast**

*Ymr1Δ* yeast cells producing the different forms of MTM1 were grown to exponential phase in selective medium and 200 $OD_{600nm}$ units were harvested at $OD_{600nm}$≈0.8. Cells lysis as well as lipids extraction and TLC separation were performed as described for the metabolic labeling. The spots corresponding to PtdIns*P* were extracted and submitted to an *in vitro* kinase assay using recombinant PtdIns5*P* 4-kinase type IIα in presence of [γ-$^{32}$P]-ATP [2,11]. Among the different PtdIns*P* species, this kinase specifically phosphorylates PtdIns5*P* to PtdIns(4,5)$P_2$. After the *in vitro* kinase reaction, lipids were separated by TLC and the spot corresponding to PtdIns(4,5)$P_2$ was scrapped off and radioactivity was measured by a scintillation counter. The measured quantity of $^{32}$P-PtdIns(4,5)$P_2$ will directly represent the *in vivo* PtdIns5*P* intracellular levels. The mass amount of PtdIns5*P* (pmol) was determined by comparison of the incorporated radioactivity in our samples with a calibration curve obtained by using di-C16-PtdIns5*P*.

**Production and purification of Adeno-Associated Virus (rAAV)**

rAAV2/1 vectors were generated by a triple transfection of AAV-293 cell line with pAAV2-insert containing the insert under the control of the CMV promoter and flanked by

serotype-2 inverted terminal repeats, pXR1 containing rep and cap genes of AAV serotype-1, and pHelper encoding the adenovirus helper functions. Cell lysates were subjected to 3 rounds of freeze/thaw, then treated with 50U/mL Benzonase (Sigma) for 30 minutes at 37°C and clarified by centrifugation. Viral vectors were purified by Iodixanol gradient ultracentrifugation followed by dialysis and concentration against DPBS using centrifugal filters (Amicon Ultra-15 Centrifugal Filter Devices 30K). Physical particles were quantified by real time PCR using a plasmid standard pAAV-eGFP and titers are expressed as viral genomes per ml (vg/ml). rAAV titers used in these experiments were 5-$7.10^{11}$ vg/ml.

**Statistical analysis**

Statistical analysis was performed using the unpaired student's *t* test unless stated otherwise. p-values of <0.05 were considered significant.

**Supplementary References**


1. Taylor GS, Dixon JE (2001) An assay for phosphoinositide phosphatases utilizing fluorescent substrates. Anal Biochem 295: 122-126.
2. Tronchere H, Laporte J, Pendaries C, Chaussade C, Liaubet L, et al. (2004) Production of phosphatidylinositol 5-phosphate by the phosphoinositide 3-phosphatase myotubularin in mammalian cells. J Biol Chem 279: 7304-7312.
3. Van Mullem V, Wery M, De Bolle X, Vandenhaute J (2003) Construction of a set of Saccharomyces cerevisiae vectors designed for recombinational cloning. Yeast 20: 739-746.
4. Parrish WR, Stefan CJ, Emr SD (2004) Essential role for the myotubularin-related phosphatase Ymr1p and the synaptojanin-like phosphatases Sjl2p and Sjl3p in regulation of phosphatidylinositol 3-phosphate in yeast. Mol Biol Cell 15: 3567-3579.
5. Gietz D, St Jean A, Woods RA, Schiestl RH (1992) Improved method for high efficiency transformation of intact yeast cells. Nucleic Acids Res 20: 1425.
6. Bonangelino CJ, Catlett NL, Weisman LS (1997) Vac7p, a novel vacuolar protein, is required for normal vacuole inheritance and morphology. Mol Cell Biol 17: 6847-6858.
7. Laporte J, Kress W, Mandel JL (2001) Diagnosis of X-linked myotubular myopathy by detection of myotubularin. Ann Neurol 50: 42-46.
8. Vida TA, Emr SD (1995) A new vital stain for visualizing vacuolar membrane dynamics and endocytosis in yeast. J Cell Biol 128: 779-792.
9. Hama H, Takemoto JY, DeWald DB (2000) Analysis of phosphoinositides in protein trafficking. Methods 20: 465-473.
10. Payrastre B (2004) Phosphoinositides: lipid kinases and phosphatases. Methods Mol Biol 273: 201-212.
11. Morris JB, Hinchliffe KA, Ciruela A, Letcher AJ, Irvine RF (2000) Thrombin

stimulation of platelets causes an increase in phosphatidylinositol 5-phosphate revealed by mass assay. FEBS Lett 475: 57-60.
